# The promoter region of *lapA* and its transcriptional regulation by Fis in *Pseudomonas putida*

**DOI:** 10.1371/journal.pone.0185482

**Published:** 2017-09-25

**Authors:** Hanna Ainelo, Andrio Lahesaare, Annika Teppo, Maia Kivisaar, Riho Teras

**Affiliations:** Chair of Genetics, Institute of Molecular and Cell Biology, University of Tartu, Tartu, Estonia; Beijing Institute of Microbiology and Epidemiology, CHINA

## Abstract

LapA is the biggest protein in *Pseudomonas putida* and a key factor for biofilm formation. Its importance and posttranslational regulation is rather thoroughly studied but less is known about the transcriptional regulation. Here we give evidence that transcription of *lapA* in LB-grown bacteria is initiated from six promoters, three of which display moderate RpoS-dependence. The global transcription regulator Fis binds to the *lapA* promoter area at six positions *in vitro*, and Fis activates the transcription of *lapA* while overexpressed in cells. Two of the six Fis binding sites, Fis-A7 and Fis-A5, are necessary for the positive effect of Fis on the transcription of *lapA in vivo*. Our results indicate that Fis binding to the Fis-A7 site increases the level of transcription from the most distal promoter of *lapA*, whereas Fis binding to the Fis-A5 site could be important for modifying the promoter area topology.

## Introduction

Bacteria, including plant-associated species from the genus *Pseudomonas*, may display two distinct lifestyles. Firstly, they can exist as freely moving planktonic cells. Secondly, given appropriate environmental conditions, they may adhere to surfaces and form complex sessile communities called biofilms. The structure of the bacterial biofilm, as well as its development, differs from species to species due to the variability of the environment and the ability of the specific bacterium to respond to various biotic and abiotic signals [1,2]. Therefore, the regulation of biofilm development is sophisticated and comprises the involvement of several global and specific regulators [2,3]. The biofilm of *Pseudomonas putida* seems to be positively regulated by availability of nutrients in LB medium. We have shown that *P*. *putida* biofilm is the strongest after 4 hours of inoculation in LB medium and it decays about threefold within the next 4 hours [4]. While exact numbers vary, similar trends of *P*. *putida* biofilm development are in agreement with earlier works [5–7].

The matrix of *P*. *putida* biofilm is proteinaceous and the two largest proteins of the bacterium, LapA and LapF, are known to play an important role in biofilm formation [4,5,8]. LapA provides cell-surface interactions [9,10] and is necessary for both for initial attachment and mature biofilm [7], whereas LapF rather provides cell-cell interactions contributing to mature biofilm formation [9]. LapA seems to be the most important biofilm factor [7,11] as no conditions efficiently rescuing the *lapA* mutant’s biofilm formation defect have been reported [5,11,12]. The second large extracellular protein, LapF, is the cell surface hydrophobicity factor [13] and probably contributes to cell-cell attachment by regulating cell hydrophobicity [9]. However, LapF seems to be an important factor for biofilm formation only when *P*. *putida* is grown in glucose minimal medium, but not in a rich medium like LB [4,9].

Although the post-translational regulation of *lapA* expression is well-described [12,14,15], the regulation of transcription has been rather briefly studied. *LapA* is transcribed from early exponential to late stationary phase and the transcription is about twice as active in the stationary phase compared to the exponential phase [16]. FleQ, c-di-GMP, Fis and the GacS-GacA two-component signal system have been shown to positively influence the expression of *lapA* [4,16–18]. The global regulator FleQ activates *lapA* transcription directly by DNA binding but its exact binding sites are yet to be determined. The activating effect of FleQ varies from 2 to 10 times between different authors and methods [16–18]. The effector molecule c-di-GMP only affects *lapA* transcription through FleQ and their effect is synergistic [17,18]. The two component system GacS-GacA regulates *lapA* mRNA levels, but whether directly or indirectly remains unknown [16]. We have shown that the global regulator Fis increases the amount of LapA 1.6 times when it is overexpressed in stationary phase *P*. *putida* [4]. However, the number and location of the *lapA* promoters remains unknown and the *in vitro* binding of regulators had not been tested prior to this work.

In *P*. *putida*, the Fis mRNA levels are highest in exponentially growing planktonic cells and drop approximately three times in stationary phase cells [19]. The pattern of Fis amount follows a surprisingly similar trend to the development of *P*. *putida* biofilm. Moreover, we have reported that artificially overexpressing Fis in the stationary phase, when the native amount of Fis is lowest, enhances the mature biofilm of *P*. *putida* 3 times in LB medium [4,20].

Fis has been shown to activate and repress the transcription of genes involved in biofilm development [21–23]. In general, Fis can affect transcription by modulating DNA supercoiling and topology (e. g., [24–26]) or directly by binding to the upstream region of genes (e. g., [22,27–29]. For example, Fis directly represses the transcription of *lapF* 4.2 times in *P*. *putida* by binding on its RpoS-dependent promoter [22]. Additionally, it has been shown that Fis can activate biofilm formation indirectly by repressing signal transduction in the *Vibrio cholerae* quorum sensing regulatory pathway [30].

Fis seems to be an essential protein for *P*. *putida* [20,31]. Fis is well described in *E*. *coli*, where the Fis-deficient strains are viable, although with a prolonged lag-phase [28]. Yet, we have been unable to construct Fis-deficient or *fis* under-expression strains in *P*. *putida* [20,31]. Therefore, a *fis* overexpression strain was used to elucidate its effects in *P*. *putida*.

The main goal of this study was to locate the promoter(s) of *lapA* and ascertain the impact of Fis on the transcription of *lapA*. In the present report, we show that the transcription of *lapA* is initiated from six promoters in LB medium, three of which display moderate RpoS-dependence. We identified six Fis binding sites in front of *lapA* by DNase I footprint and gel-shift assays. Two of the six binding sites are necessary for the Fis-enhanced expression of *lapA in vivo*. Surprisingly, these two binding sites are located 575 bp and 755 bp upstream of the *lapA* start codon. Fis activates the transcription of *lapA* from the most distal promoter P_*lapA8*_ and can additionally regulate *lapA* transcription via modification of *lapA* upstream DNA topology.

## Materials and methods

### Bacterial strains, plasmids, oligonucleotides and media

The bacterial strains and plasmids used in this study are described in S1 Table and oligonucleotides in S2 Table. *E*. *coli* was incubated at 37°C and *P*. *putida* at 30°C. *E*. *coli* strain DH5αλ*pir* [32] was used for cloning suicide vector pEMG constructs and *P*. *putida* PSm for routine cloning pBLKT and p9TT_B_lacZ constructs [22,33]. Bacteria were grown in LB medium [34]. Solid media contained 1.5% Difco agar. Antibiotics were added at the following concentrations: gentamicin, 10 μg ml^-1^; kanamycin, 50 μg ml^-1^; benzylpenicillin, 1.5 mg ml^-1^; streptomycin, 200 μg ml^-1^. Bacteria were electrotransformed as described by Sharma & Schimke [35].

### DNA manipulations and strains construction

**Fig 1:**
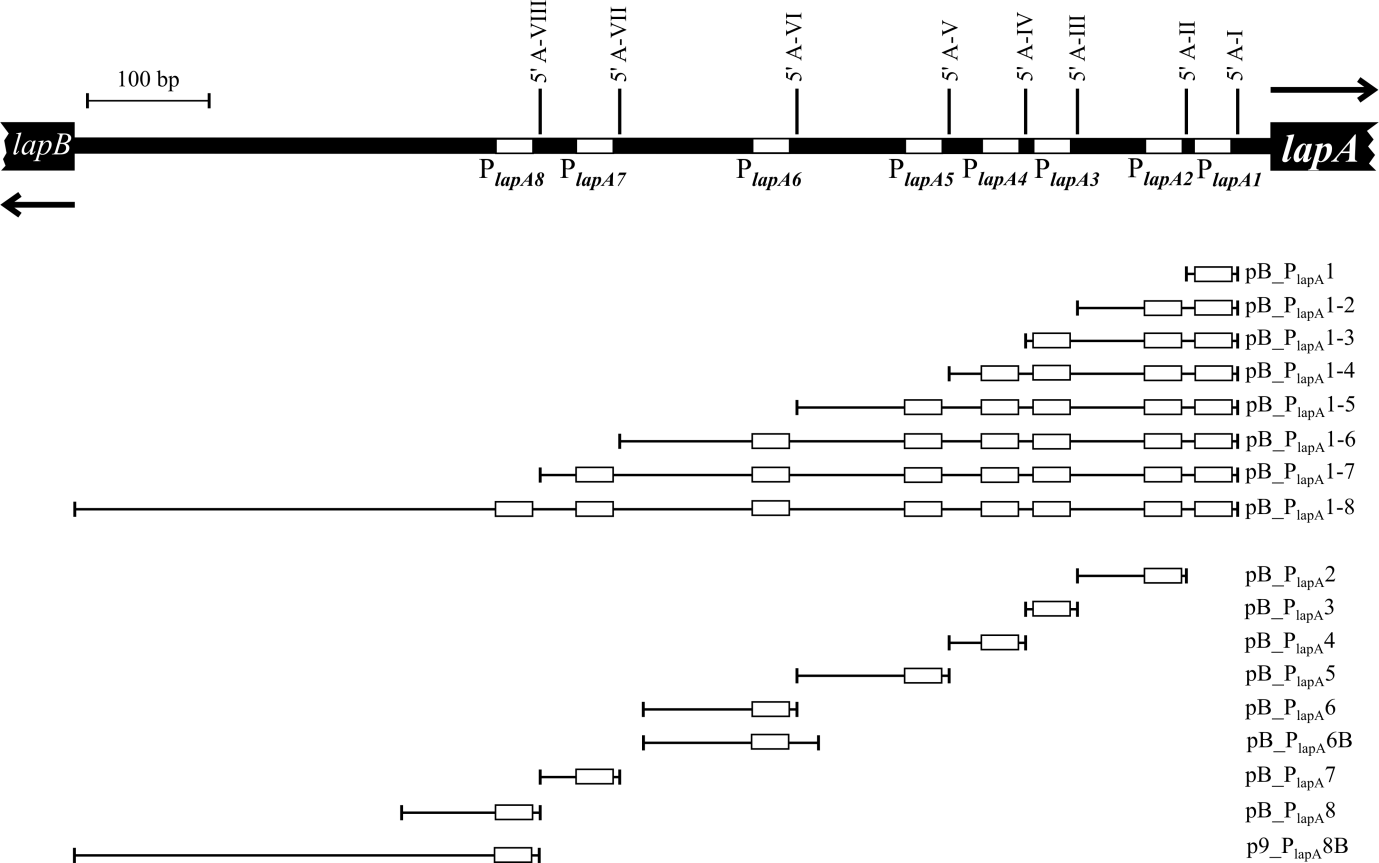
Scheme of the *lapA* promoter region, promoter region fragments cloned into pBLKT (or p9TT_B_lacZ) plasmid and the names of yielding constructs. The first nucleotides of the mRNA 5ʹ ends determined by 5ʹ RACE are written in bold and designated as A-I to A-VIII. Potential promoters P_*lapA1*_ to P_*lapA8*_ are highlighted as white boxes in the black DNA. The black arrows show the beginnings of *lapA* and *lapB* genes.

Two types of promoter probe vectors were used to clone *lapA* promoter fragments in front of the *lacZ* gene and thereafter measure β-galactosidase activity. A medium-copy number pBRR1-based promoter probe vector pBLKT [22,36] was used for the functionality assessment of potential promoters (S1 Table). A low-copy-number RK2-based promoter probe vector p9TT_B_lacZ [33,37] was used to ascertain the influence of Fis to the transcription of *lapA* (S1 Table). The *lapA* promoter regions were amplified by PCR, and all fragments were cloned into the pBLKT or p9TT_B_lacZ BamHI site except for P_*lapA8*_, which was cloned into blunted BamHI site (Fig 1). The oligonucleotides, the length of amplified *lapA* promoter regions and content of DNA used for construction of plasmids are specified in the S1 and S2 Tables.

For the construction of plasmids carrying substitutions in *lapA* promoter region (mutated Fis binding sites and mutated -10 box of potential promoters), site-directed mutagenesis of wild-type *lapA* promoter region was performed using two sequential PCRs and the *P*. *putida* PSm chromosomal DNA or different plasmids as a template. In the first PCR, the substitutions were introduced into the target site using a pair of oligonucleotides, one of which contained substituted nucleotides in the target site. In the second PCR, the needed *lapA* promoter region was amplified by using the second pair of oligonucleotides, one of which was the product of the first PCR. All mutated PCR fragments were cloned into the BamHI site of pBLKT or p9TT_B_lacZ plasmids [22,33].

For construction of pBP_lapA_1-8_F1mut and F2mut, respective mutational primers LapA-1mut-uus or LapA-2mut and LapA-I-rev were used for first PCR. The products of first PCR and PP0167-down were used as oligonucleotides for the second PCR to amplify a 951 bp long *lapA* promoter area. For construction of pBP_lapA_1-8_F4mut, F5mut, F6mut and F7mut, the respective mutational primers LapA-4mut-uus, FisA5-mut, FisA6-mut or FisA7-mut and PP0167 down were used for first PCR and the products of the first PCR and LapA-I-rev for second PCR. These amplifications resulted in fragments with substituted nucleotides in the different Fis binding sites but are otherwise identical to p9_P_lapA_1-8. The number of substitutions made in every mutated Fis binding site is specified in S1 Table. For the construction of p9_P_lapA_1-8_F1,2mut, the plasmid p9_P_lapA_1-8_F2mut was used as the template and mutations of Fis-A1 binding site were introduced as described previously via two sequential PCRs. For the construction of p9_P_lapA_1-8_F4,5,6,7mut, plasmids p9_P_lapA_1-8_F4,5mut and p9_P_lapA_1-8_F6,7mut were constructed first. The construct p9_P_lapA_1-8_F4mut was used as the template and mutations of the Fis-A5 were introduced to make p9_P_lapA_1-8_F4,5mut. Additionally, p9_P_lapA_1-8_F6mut was used as the template and Fis-A7 was mutated via two sequential PCRs to make p9_P_lapA_1-8_F6,7mut. Thereafter, p9_P_lapA_1-8_F6,7mut was amplified with FisA6-mut and PP0167-down primers. NheI (Thermo Scientific) digestion was used to cut the template p9_P_lapA_1-8_6,7m in the mixture of first PCR to hinder its use as a template in the second PCR. The product of first PCR, oligonucleotide lapA-I-rev and template p9_P_lapA_1-8_4,5m were used in the second PCR.

For construction pB_P_lapA_1-3 variant carrying the *lapA* promoter area with substitutions in the potential -10 box of P_*lapA3*_, two sequential PCR-s were used. The substitutions in the -10 box of the potential promoter P_*lapA3*_ was introduced by an oligonucleotide LapA-IIImut-piken in the first PCR. LapA-IV was used as the second oligonucleotide in the PCR mixture and pB_P_lapA_3mut as a template. The PCR product carrying the mutated -10 box of P_*lapA3*_ was obtained. The second PCR was carried out with a pair of oligonucleotides, one of which was the product of the first PCR and the second was LapA-I-rev and p9_P_lapA_1-8 as template (S1 and S2 Tables).

For construction of p9TT_B_lacZ variants carrying the individual promoters P_*lapA6*_, P_*lapA7*_, P_*lapA8*_ and functional Fis binding site(s) or mutated Fis binding site(s), the *lapA* promoter region was amplified by PCR using oligonucleotides shown in S1 Table and different templates. The pB_P_lapA_1-8 was used as a PCR template for the construction of p9_P_lapA_6B, p9_ P_lapA_7 and p9_ P_lapA_8B; the p9_A1-8_F4mut for construction of p9_P_lapA_6B_F4mut; the p9_P_lapA_1-8_F5mut for construction of p9_P_lapA_7_F5mut; and the p9_P_lapA_1-8 _F6mut, p9_P_lapA_1-8_F7mut and p9_P_lapA_1-8_F4567mut for construction of p9_P_lapA_8B _F6mut, p9_P_lapA_8B_F7mut and p9_P_lapA_8B_F67mut, respectively. Additionally, the parallel constructs pB_P_lapA_6B and pB_P_lapA_6B_4mut were created similarly to p9TT_B_lacZ variants.

For construction of PSm Δ*rpoS*, the DNA regions which flanked the *rpoS* were cloned into the suicide vector pEMG using the protocol described by Martinez-Garcia *et al*. [32]. The 457 bp long region located upstream of *rpoS* (Up) was amplified by primers RpoS-I-fw and RpoS-I-rev and the 468 bp long region downstream of *rpoS* (Down) was amplified by primers RpoS-2-fw and RpoS-2-rev. The Up and Down *rpoS* flanking regions were joined together by overlap extension (SOE)-PCR [38]. Thereafter the 925-bp PCR fragment (Up+Down) was purified and cloned into pEMG using the *Eco*RI and *Bam*HI sites, resulting in pEMG-ΔrpoS (S1 Table). The *rpoS* deletion mutant of *P*. *putida* strain PSm (S1 Table) was constructed by a previously described protocol [32]. The pEMG-ΔrpoS was delivered to *P*. *putida* PSm by electroporation [39] to obtain a cointegrate between *P*. *putida* chromosome and pEMG-ΔrpoS carrying the recombination targets. A mutant with desired deletions was obtained after electroporating bacteria with plasmid pSW and expression of the Sce-I homing restrictase from the pSW plasmid [40].

All designed constructs were sequenced in order to exclude PCR-generated errors in the cloned DNA fragments. The accuracy of recombination was checked by sequencing the relevant regions of *P*. *putida*’s chromosome.

### Identification of 5ʹ ends of mRNA by RACE

The mRNA 5ʹ ends of the *lapA* gene were identified by RACE (rapid amplification of cDNA ends) as described by Sambrook and Russell [41]. The cells were grown in LB medium for 18 hours at 30°C and total RNA was purified using the Thermo Scientific GeneJET RNA Purification kit. To obtain RNA from exponentially growing bacteria, cells were pre-grown for 18 hours, diluted 50 times and then grown to the optical density of 0.5. The cells were thereafter diluted 1:1 and grown for 30 minutes. This step was repeated 3 times. After the third time the cells were grown to the optical density of 0.5. 1.5 μg of purified total RNA and the LapA-RACE1 primer were used for the synthesize the first strand of cDNA. The second strands of cDNA were synthesized by primers Adapt-pikkC or Adapt-pikkT, with 5ʹ ends binding accordingly to poly-G or poly-A, synthesised by terminal deoxynucleotidyltransferase (TdT) to the 3ʹ ends of the first strand of cDNA. To amplify the second strand of cDNA, the Adapt-lyh and LapA-RACE2, LapA-rev, LapA-IV-rev or LapA-VI-rev primers were used. Zymo Research DNA Clean & Concentrator^TM^-5 kit was used for DNA purification between RACE steps.

### Prediction of Fis binding sites on the promoter region of the *lapA* gene

Putative Fis binding sequences on the promoter regions of the *lapA* gene were predicted using the *E*. *coli* Fis binding sites matrix [42] and the matrix-scan program available at the Regulatory Sequence Analysis Tools homepage [43]. The -1000 bp to -1 bp DNA region of the *lapA* gene was used for the prediction of potential Fis binding sites. The Markov model of one order, organism-specific probability of nucleotides in the upstream region of genes in *P*. *putida* KT2440 and a *P*-value upper threshold of 0.001 were selected for the conditions of the background model. The rest of the parameters were left at the program’s default values.

### DNase I footprint analyses

DNase I footprint assays were performed for the identification of *P*. *putida* Fis binding sequences on the *lapA* promoter region. PCR-amplified fragments were used for DNase I footprint assay and were generated as follows. To study Fis binding to Fis-A1 and Fis-A2 sites, 207 bp-long DNA fragments containing the two sites were amplified by primers LapAdown and LapA2up. Depending on the template (p9_P_lapA_1-8, p9_P_lapA_1-8_F1mut or p9_P_lapA_1-8_F2mut), the fragments contained the wild-type Fis-A1 and Fis-A2 sites or either the mutated Fis-A1mut site or the mutated Fis-A2mut site. The 220 bp-long Fis-A4 fragment was amplified with LapAdown2 and LapA-fw using the full-length *lapA* promoter construct with or without mutated Fis-A4 binding site as template. To study Fis binding to Fis-A5 and Fis-A6 sites, the 235 bp-long DNA fragment containing the two sites was amplified using PP0167-I-fw and PP0168-I-fw primers. p9_P_lapA_1-8, p9_P_lapA_1-8_F5mut or p9_P_lapA_1-8_F6mut were used as templates. The 238 bp-long Fis-A7 fragment was amplified with LapBCup and LapBCdown using the full-length *lapA* promoter construct with or without mutated Fis-A7 binding site as template. Primers used for amplifications are listed in S2 Table. The following procedures: labelling PCR products with [γ-^32^P]-ATP, preparing reaction mixtures and gel electrophoresis were carried out as described by Teras *et al*. in 2009.

### Gel mobility shift assay

The same radiolabelled PCR products that were used for DNase I footprint assays were used for the gel mobility assay. Additionally, the non-labelled PCR product containing the Fis binding site LF2 [44] and a PCR product without Fis binding site RF1 [44] were used in out-competition experiments. The unlabelled DNA fragment LF2 was amplified using the oligonucleotides TnLsisse and SIDD-2. Oligonucleotides PRH8 and Tnots were used for the amplification of unlabelled DNA RF1. Plasmids pLA1-12 and pRA1-12 [44] were used as templates for amplifying LF2 and RF1, respectively. Amounts of competing DNA in the reaction mixes were calculated in molecules. Binding reactions with purified *P*. *putida* His-tagged Fis were carried out with 2 × 10^10^ molecules (750–1000 c.p.m.) of labelled DNA fragment in a reaction buffer (24 mM Tris/HCl pH 7.5, 50 mM KCl, 10 mM MgCl_2_, 1 mM CaCl_2_, 0.1 mM EDTA, 5% glycerol, 0.05 μg BSA μl^-1^ and 0.05 μg salmon sperm DNA μl^-1^) in a final volume of 20 μl. The mixtures were preincubated for 20 min at room temperature. After incubation, reaction mixtures were applied to a 5% non-denaturing polyacrylamide gel buffered with TBE (50 mM Tris, 60 mM boric acid, 5 mM EDTA; pH 7.5). Electrophoresis was carried out at 4°C at 10 V cm^-1^ for 3 hours. Gels were vacuum dried and exposed to a Typhoon Trio screen (GE Healthcare).

### Measurement of β-galactosidase activity

To measure β-galactosidase activities, the pBLKT or p9TT_B_lacZ constructs containing the *lapA* promoter region in front of *lacZ* gene were electroporated into *P*. *putida* wild-type strain PSm or IPTG-inducible *fis* overexpression strain F15. The resulting colonies were streaked onto LB agar plates, grown overnight at 30°C and incubated at 4°C for 3 days. Incubation at 4°C reduced the variability between biological replicates. The cells were thereafter grown in LB medium with or without 1 mM IPTG supplementation for 18 hours at 30°C. 18 hours of incubation was chosen as the cells are in stationary phase after 18 hours of growth and the growth rate difference between PSm and F15 does not play a role [20]. Also the native amount of Fis has dropped at that timepoint [19]. For exponential phase measurements, cells were pre-grown for 18 hours, diluted 50 times and then grown to the optical density of 0.5. The method of serial dilutions for growing exponential phase bacteria [16] enabled similar results of *lapA* transcription activity compared to a single dilution (data not shown), indicating that one dilution is enough for exponential phase measurements. The β-galactosidase measurements from cell suspension were performed according to the protocol of Miller [34]. At least five independent measurements were performed.

### Statistical analysis

Factorial analysis of variance (ANOVA) and *post-hoc* Bonferroni test at a significance level of 0.05 were used to assess the variability of experimental data. The calculations were performed using Statistica 13 software.

## Results

### Mapping *lapA* promoters

**Fig 2:**
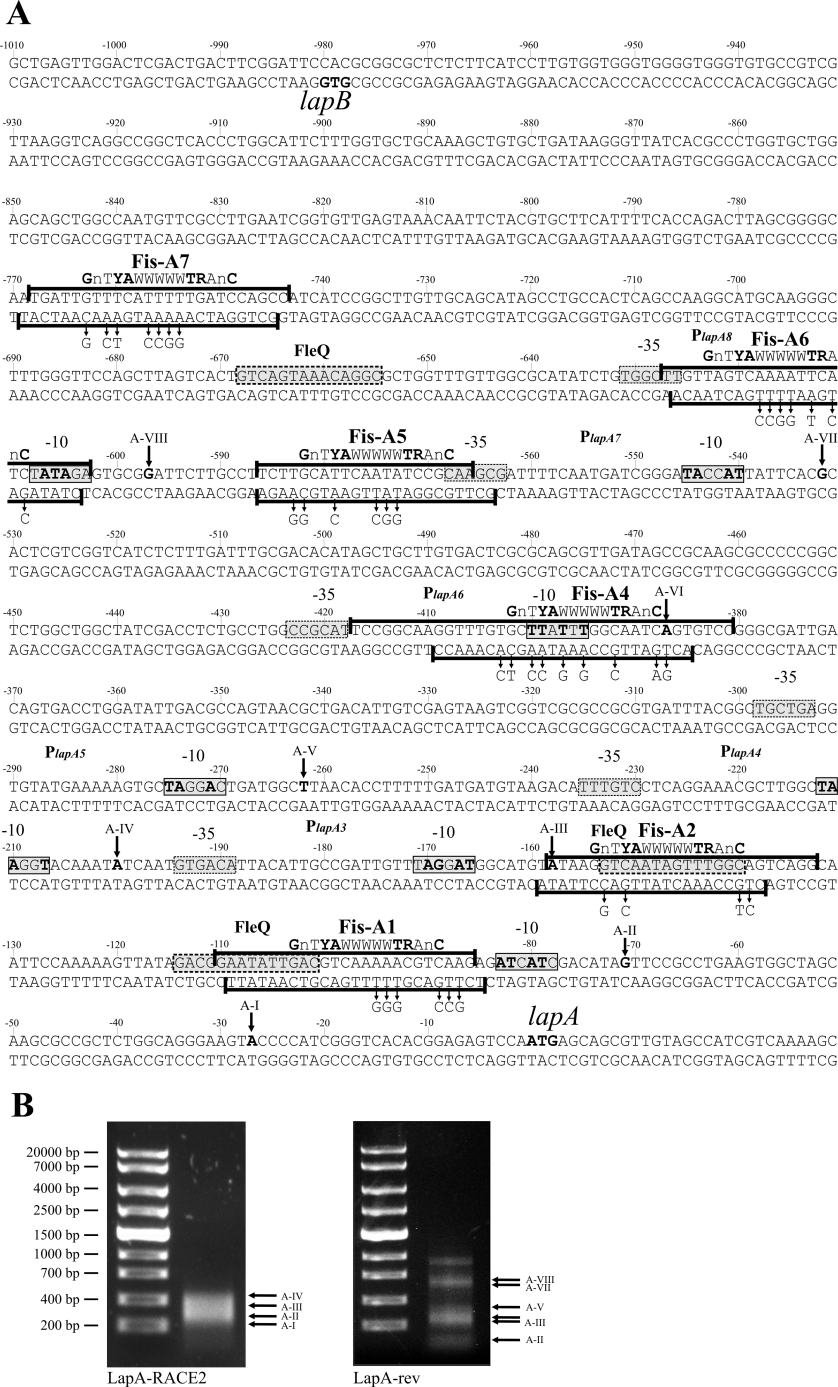
Mapped mRNA 5ʹ ends, promoters and Fis binding sites at the *lapA* promoter region. (A) Sequence of the *lapA* promoter region and downstream DNA. The start codons of the *lapA* and *lapB* genes are shown in bold. The first nucleotides of the mRNA 5ʹ ends determined by 5ʹ RACE are written in bold and designated as A-I to A-VIII. The potential –10 and –35 elements of the *lapA* promoters are shown in grey boxes with solid and dotted outlines, respectively. The Fis binding sites Fis-A1, Fis-A2 and Fis-A4 to Fis-A7 are shown in black brackets. The *E*. *coli* Fis binding consensus according to Finkel and Johnson (1992) and Shao *et al*. (2008) is shown above every Fis binding sequence [46,47]. The most important nucleotides in the *E*. *coli* Fis binding consensus are shown in bold. The point mutations in the Fis binding sequences are indicated by arrows on the antisense strand. The substituted nucleotides in the -10 boxes of potential promoters are shown in bold on the sense strand. *In silico* predicted FleQ binding sequences [48] are indicated by grey boxes. (B) Agarose gel electrophoresis of cDNA amplified by the RACE method for the identification of the *lapA* mRNA 5ʹ ends. The arrows point to the PCR products used to determine the mRNA 5ʹ ends. The primer used is shown under the gel images.

In order to investigate where the promoters of *lapA* are located, we mapped the 5ʹ ends of *lapA* mRNA obtained from exponential and stationary phase LB-grown *P*. *putida* by RACE. Eight 5ʹ ends for the *lapA* mRNA located at 27, 71, 158, 200, 262/3, 387, 532/3 and 597 bp upstream of the *lapA* start codon were identified from stationary phase cells (Figs 1 and 2). The ninth PCR product, approximately 900 bp long (Fig 2B, above A-VIII), was probably the result of a nonspecific amplification since it was not verified as a 5ʹ end of *lapA* mRNA by cDNA sequencing. The same 5ʹ ends of *lapA* mRNA were identified from exponentially growing bacteria, except for the 5ʹ ends located at 27, 262 and 597 bp upstream of the *lapA* start codon. Using the consensus sequence of *E*. *coli* sigma70-dependent promoters [45] we predicted the -10 boxes of the eight putative promoters P_*lapA1*_ to P_*lapA8*_ (Figs 1 and 2).

To confirm that the identified 5ʹ mRNA ends correspond to transcription start sites, *lapA* promoter region fragments were cloned into the promoter probe vector pBLKT containing the *lacZ* reporter gene (Fig 1). All of the cloned DNA fragments shared a common 3ʹ end at the position -27 from the *lapA* gene start codon. The first construct pB_P_lapA_1 contained one potential promoter P_*lapA1*_ (Fig 1). The second construct pB_P_lapA_1-2 contained two potential promoters P_*lapA1*_ and P_*lapA2*_ and so on; so that each subsequent construct contained one additional potential promoter compared to the previous one. The β-galactosidase activity was measured in stationary-phase cells of the *P*. *putida* wild-type strain PSm (Table 1). By extending the *lapA* promoter region from the 5ʹ end, we expected to see an increase in β-galactosidase activity every time a functional promoter was added.

**Table 1:** B-galactosidase activity (Miller units) expressed from different length *lapA* promoter constructs.

Construct	4 h		18 h	
	PSm	PSmΔrpoS	PSm	PSmΔrpoS
pBLKT*	0.36 (0.05) a	0.58 (0.30) a	0.98 (0.05) b	1.09 (0.13) b
pB_P_lapA_1	*-*	*-*	0.8 (0.2) a	*-*
pB_P_lapA_1-2	*-*	*-*	9.9 (2.5) a	*-*
pB_P_lapA_1-3	*-*	*-*	570.6 (46.4) b	*-*
pB_P_lapA_1-4	*-*	*-*	501.5 (95.7) bc	*-*
pB_P_lapA_1-5	*-*	*-*	567.7 (21.4) bc	*-*
pB_P_lapA_1-6	*-*	*-*	861.7 (53.0) d	*-*
pB_P_lapA_1-7	*-*	*-*	457.5 (95.4) c	*-*
pB_P_lapA_1-8	*-*	*-*	516.7 (37.7) bc	*-*
pB_P_lapA_2	9.7 (1.6) a	7.7 (1.0) a	18.4 (3.3) b	16.9 (2.4) b
pB_P_lapA_2mut	7.4 (1.2) a	*-*	15.1 (3.0) b	*-*
pB_P_lapA_3	398.4 (31.2) b	480.2 (44.0) b	1217.8 (106.5) c	1443.3 (90.0) d
pB_P_lapA_3mut	28.7 (2.6) a	*-*	71.0 (4.4) a	*-*
pB_P_lapA_4	70.4 (9.0) b	67.7 (3.2) b	374.5 (74.2) c	385.5 (113.8) c
pB_P_lapA_4mut	2.4 (0.6) a	*-*	10.8 (2.1) ab	*-*
pB_P_lapA_5	20.9 (2.3) b	22.6 (4.1) b	91.2 (5.5) d	83.9 (9.0) d
pB_P_lapA_5mut	10.2 (0.8) a	*-*	38.8 (2.9) c	*-*
pB_P_lapA_6	8.1 (1.1) b	8.7 (1.3) b	34.4 (1.5) d	19.7 (1.5) c
pB_P_lapA_6mut	0.4 (0.1) a	*-*	2.4 (0.3) a	*-*
pB_P_lapA_7	8.8 (1.5) b	7.6 (0.3) b	37.6 (4.0) d	24.0 (1.5) c
pB_P_lapA_7mut	1.1 (0.1) a	*-*	2.8 (0.3) ab	*-*
pB_P_lapA_8	32.6 (2.2) b	35.9 (2.4) b	163.2 (7.5) d	123.0 (11.5) c
pB_P_lapA_8mut	12.0 (1.4) a	*-*	33.1 (7.4) b	*-*

*The promoterless pBLKT does not contain *lapA* upstream DNA.

Data from at least 5 independent measurements is shown. 95% confidence intervals are shown in parentheses. Not determined activities are marked with the “-”symbol. Groups of data analysed separately are divided by horizontal lines. Letters a-e depict different homogeneity groups according to ANOVA *post hoc* Bonferroni test. Identical letters denote non-significant differences (*P*>0.05) between averages of β-galactosidase activity.

All constructed plasmids ensured β-galactosidase activity in *P*. *putida* wild-type strain PSm except pB_P_lapA_1, which β-galactosidase activity was comparable to the activity of a promoterless pBLKT vector which was approximately 1 Miller unit (Table 1). This indicated that there is no σ^70^ type promoter in pB_P_lapA_1. The wild-type cells harbouring pB_P_lapA_1-2 showed a β-galactosidase activity of 10 Miller units. The increase in β-galactosidase activity compared to pB_P_lapA_1 was not statistically significant, suggesting that this upstream *lapA* region also lacks a functional σ^70^ type promoter or contains only a very weak one. Only two constructs, pB_P_lapA_1-3 and pB_P_lapA_1-6, displayed a statistically significant increase in β-galactosidase activity compared to the previous shorter version, suggesting that P_*lapA3*_ and P_*lapA6*_ are *lapA*’s promoters (Table 1). Adding subsequent hypothetical promoters P_*lapA4*_, P_*lapA5*_, P_*lapA7*_ and P_*lapA8*_ did not increase the β-galactosidase activity, which suggested that these may not contribute to transcriptional activation of *lapA*. However, cumulative extension of the regulatory region may add potential regulator (repressor) binding sites in addition to promoters. Thus, it might be possible that additional regulators that bind longer fragments may mask the effect of weaker promoters in these constructs.

Therefore, to examine this possibility, we decided to assess the effect of each individual potential promoter to the transcription of the reporter gene (Table 1). The β-galactosidase activity of hypothetical promoters P_*lapA2*_, P_*lapA3*_, P_*lapA4*_, P_*lapA5*_, P_*lapA6*_, P_*lapA7*_ and P_*lapA8*_ cloned into the promoter probe vector pBLKT were measured in both exponential and stationary-phase cells of the *P*. *putida* wild-type strain PSm (Table 1). All assessed pBLKT constructs (except for previously measured pB_P_lapA_1) revealed β-galactosidase activity in PSm (Table 1). To verify the existence of promoters in cloned *lapA* upstream region, we used the same individual promoter regions but with mutated -10 boxes (Table 1). Although the number of substituted nucleotides varied, in general, the A-T nucleotides of the σ^70^ type promoter consensus were substituted with G-C nucleotides. Disrupting putative -10 boxes decreased the activity of promoters P_*lapA3*_, P_*lapA4*_, P_*lapA6*_ and P_*lapA7*_ between 8 and 35 times in both stationary phase and exponentially growing cells (Table 1), confirming that these are functional promoters. Disrupting -10 boxes of putative promoters P_*lapA5*_ and P_*lapA8*_ reduced the LacZ activity in both growth phases between 2 and 5 times (Table 1), showing that these are probably functional promoters as well. At the same time, mutating the potential -10 box of P_*lapA2*_ had no effect on β-galactosidase activity in stationary phase nor in exponentially growing PSm (Table 1). This indicated that P_*lapA2*_ is either not a σ^70^ promoter or not a functional promoter at all.

**Fig 3:**
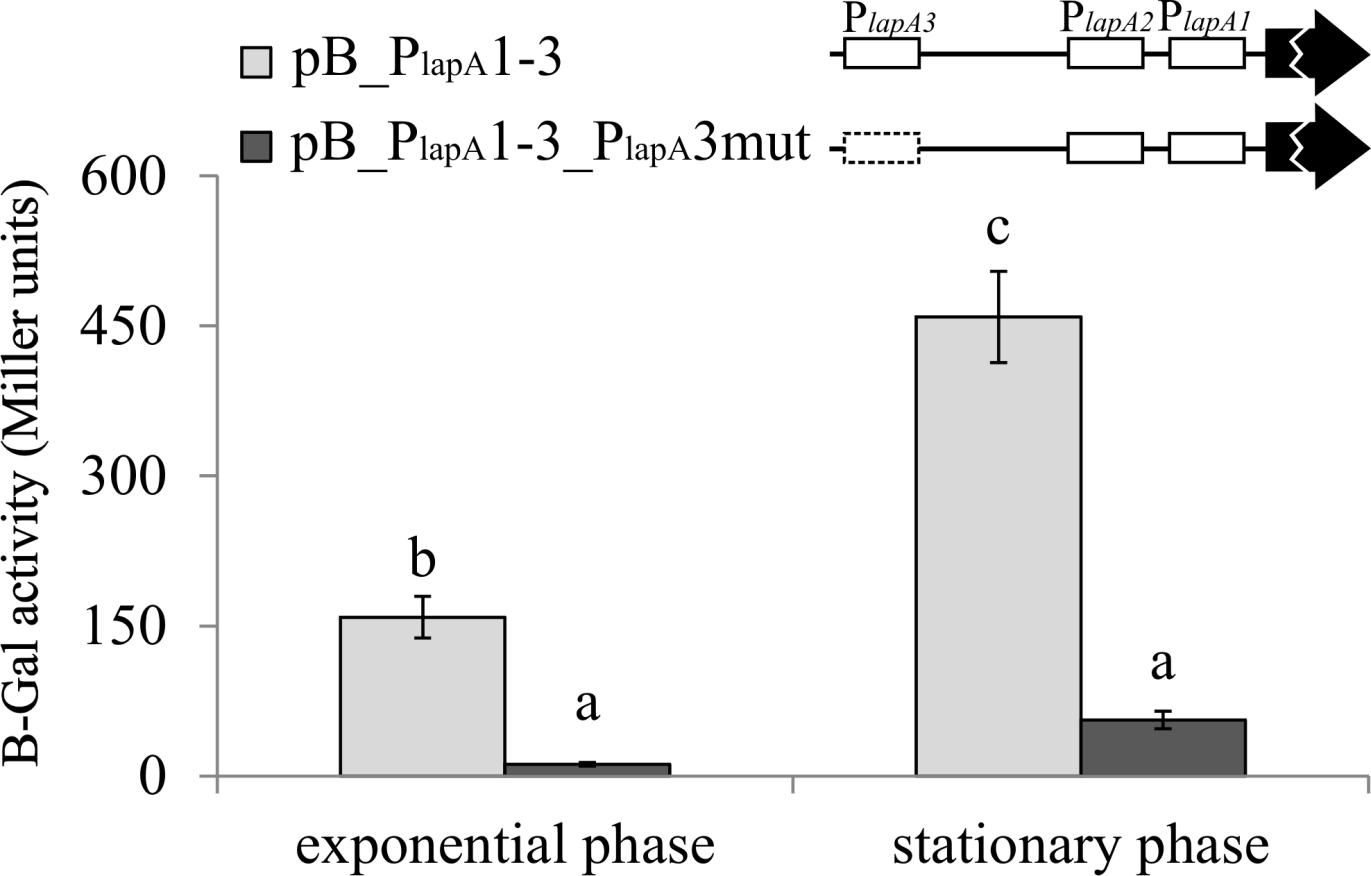
The effect of mutating P_*lapA3*_ on the transcription from the proximal upstream DNA of *lapA*. B-galactosidase (β-Gal) activity expressed from the *lapA* promoter-*lacZ* reporter constructs containing upstream DNA at position -27 to -200 was measured in *P*. *putida* wild-type strain PSm grown in LB medium to optical density of 0.5 (exponential phase) and for 18 hours (stationary phase). Schemes of *lapA* proximal upstream DNA carrying the functional P_*lapA3*_ promoter and predicted promoters P_*lapA1*_ and P_*lapA2*_ are shown above the diagrams. The Dotted box denotes the mutated promoter P_*lapA3*_, *lacZ* reporter gene is shown as a black arrow and location of sequences previously described as P_*lapA1*_ and P_*lapA2*_ in white boxes. Vertical bars denote 95% confidence intervals of means. Data of at least 9 independent measurements is shown. Letters a–c depict homogeneity groups according to ANOVA *post hoc* Bonferroni test. Identical letters denote non-significant differences (*P*>0.05) between averages of β-galactosidase activity.

To examine the possibility of the presence of any unidentified promoters in the proximal upstream region of *lapA* gene, we constructed pB_P_lapA_1-3_P_lapA_3mut (S1 Table). This construct is otherwise identical to pB_P_lapA_1-3 except for the mutated -10 box of P_*lapA3*_, which was identified as the strongest *lapA* promoter (Table 1). *P*. *putida* PSm harbouring pB_P_lapA_1-3 with mutated P_*lapA3*_ showed a LacZ activity of 14 Miller units in exponential growth phase and 62 Miller units in stationary phase, which is respectively 12.6 and 7.1 times less than wild-type pB_P_lapA_1-3 (Fig 3). The LacZ activity measured with pB_P_lapA_1-3_P_lapA_3mut was probably a residual promoter activity left after mutating P_*lapA3*_ because the mutated P_*lapA3*_ promoter revealed a similar pattern in the study of individual promoters (Table 1). Thus, it seems that P_*lapA3*_ is the most important promoter for *lapA* transcription in LB medium and the proximal region of *lapA* up to P_*lapA3*_ does not carry any additional promoters that would be active in LB medium, at least not at a considerable level.

### RpoS-dependency of *lapA* promoters

We were interested in the RpoS-dependency of all identified promoters. Considering that *lapA* has six promoters unequally contributing to the expression of *lapA*, we decided to test the RpoS-dependency of all promoters individually to avoid the masking effects of strong promoters like P_*lapA3*_. We measured β-galactosidase activity in stationary phase PSmΔrpoS carrying the individual promoter constructs (Table 1). This experiment revealed that P_*lapA6*_, P_*lapA7*_ and P_*lapA8*_ show a moderate RpoS-dependence in stationary phase as the plasmids with the corresponding promoters ensured decreased β-galactosidase activity in *rpoS* deletion strain compared to the β-galactosidase activity in wild-type strain (Table 1). P_*lapA4*_ and P_*lapA5*_ are not RpoS-dependent and P_*lapA3*_ showed an approximately 1.2 times higher activity in the *rpoS* deletion strain than in the wild-type PSm (Table 1).

To verify the RpoS-dependence of promoters, we measured the β-galactosidase activity also in exponentially growing PSm and PSmΔrpoS (Table 1). The transcription and translation of *rpoS* in *P*. *putida* is downregulated in exponentially growing cells [19,49,50] and therefore RpoS should not affect promoters in exponentially growing cells [9,51,52]. As expected, *rpoS* deletion has no statistically significant effect on the activities of the measured promoters P_*lapA3*_, P_*lapA4*_, P_*lapA5*_, P_*lapA6*_, P_*lapA7*_ and P_*lapA8*_ in exponentially growing cells. However, it seems that RpoS did not affect the activity of P_*lapA3*_, since similarly to stationary phase cells, exponentially grown cells carrying pB_P_lapA_3 exhibited an approximately 1.2-times higher β-galactosidase activity in the absence of RpoS. Moreover, Martinez-Gil *et al*. have reported in 2014 that the transcription from promoter probe vector carrying *lapA* proximal promoters identified in this work as P_*lapA3*_, P_*lapA4*_ and P_*lapA5*_ is RpoS-independent. Thus, the statistically significant activation of P_*lapA3*_ promoter in PSmΔrpoS strain can be a type I error of statistical hypothesis testing without biological importance.

Altogether, we have identified six *lapA* promoters with recognisable -10 elements: P_*lapA3*_, P_*lapA4*_, P_*lapA5*_, P_*lapA6*_, P_*lapA7*_ and P_*lapA8*_. All of them are active in both exponential and stationary phase. P_*lapA3*_ seems to be the most important for the expression of *lapA* as it shows the highest activity in both β-galactosidase experiments: cumulative extensions of the 5ʹ end of *lapA* promoter area and studying individual promoters. P_*lapA6*_, P_*lapA7*_ and P_*lapA8*_ display RpoS-dependence. As the effects are moderate, these promoters are probably recognized by both sigma factors RpoS and RpoD.

### Mapping Fis binding sites in the *lapA* promoter region

We have previously reported that *fis*-overexpression increases the amount of LapA in *P*. *putida* about 1.6 times [4]. However, it was yet unclear if Fis regulates the expression of *lapA* directly by binding *lapA* promoter area and controlling its transcription. To elucidate where Fis binding sites are located, we used *in silico* prediction and thereafter determined Fis binding by DNase I footprint and gel-shift analysis.

**Fig 4:**
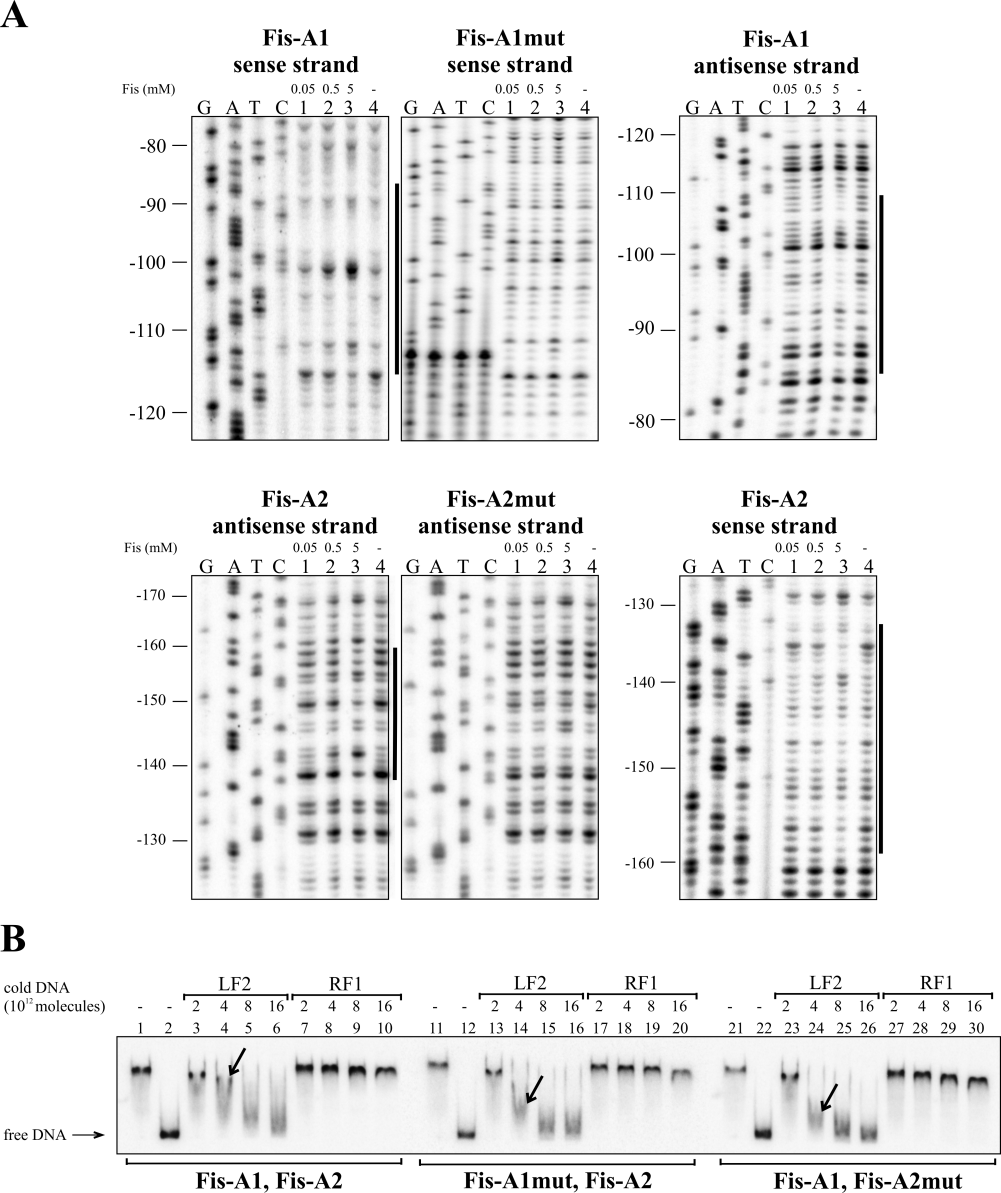
Fis binding to Fis-A1 and Fis-A2 sites upstream of the *lapA* gene. (A) Protection of the *lapA* upstream DNA against DNase I cleavage by Fis binding on the sense and the antisense strands. Lines at the right side of the panels indicate the regions protected by Fis from DNase I cleavage at the positions -110 to -85 on the sense strand and -109 to -84 on the antisense strand corresponding to Fis-A1; and -158 to -132 on the sense strand and -159 to -138 on the antisense strand corresponding to Fis-A2. (B) Gel shift assay of the Fis binding to the *lapA* promoter DNA containing the wild-type Fis binding site Fis-A1 and Fis-A2 and one or the other mutated site. 2 × 10^10^ molecules of radioactively labelled PCR products containing both Fis binding sites Fis-A1 and FisA2 or one mutated binding site FisA1mut-Fis-A2, and Fis-A1-FisA2mut were used in Fis binding assay. Fis was outcompeted from Fis-DNA complex with unlabelled PCR product containing the Fis binding site (LF2) or a PCR product without Fis binding site (RF1). Arrows point to different dissociation of Fis from radioactively labelled DNA in favour of binding unlabelled Fis-specific DNA. Added unlabelled DNA was calculated in molecules. 0.46 μM Fis was used in each reaction mixture except mixtures without Fis in lanes 2, 12 and 22.

**Fig 5:**
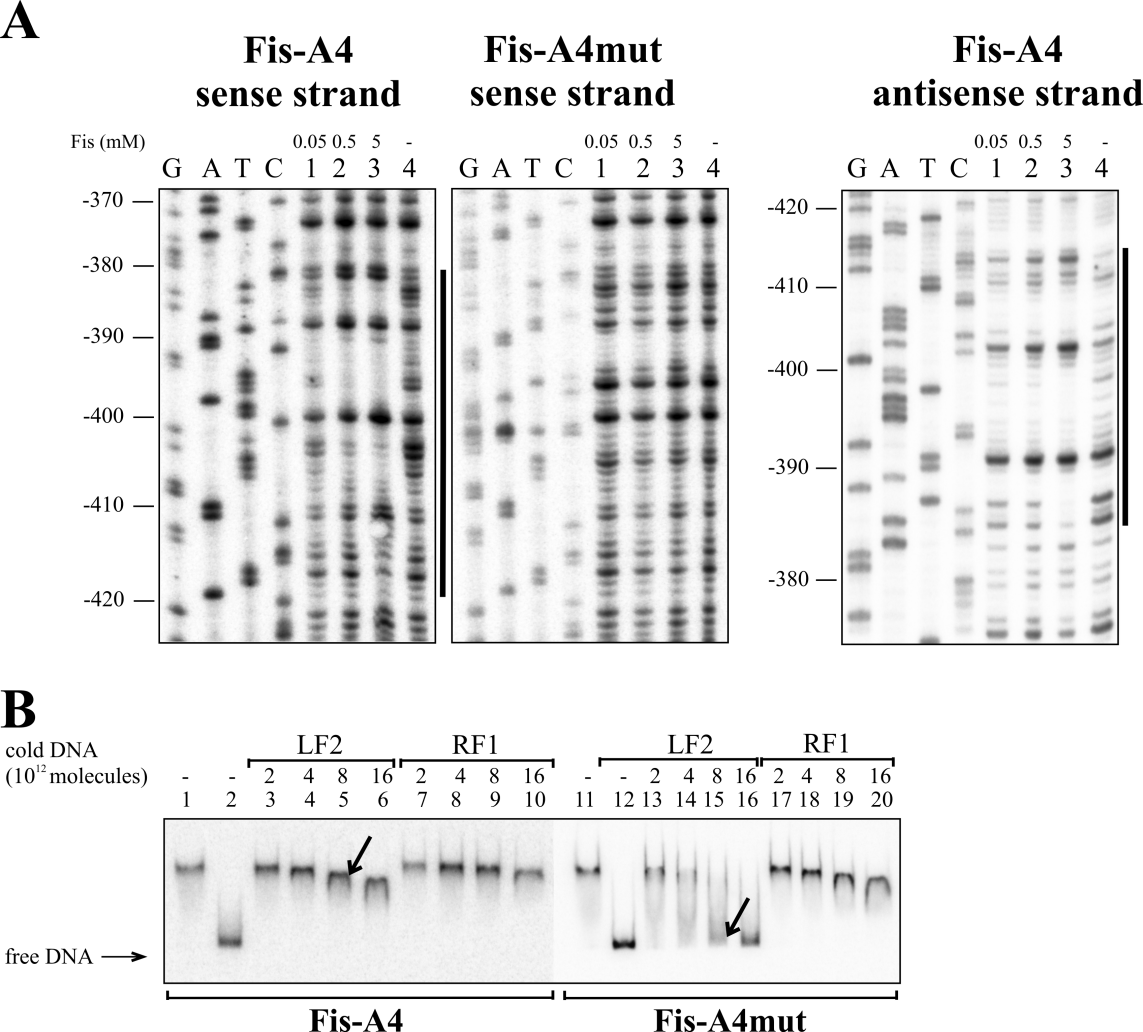
Fis binding to Fis-A4 site upstream of the *lapA* gene. (A) Protection of the *lapA* upstream DNA against DNase I cleavage by Fis binding on the sense and the antisense strands. Lines at the right side of the panels indicate the regions protected by Fis from DNase I cleavage at the positions -417 to -381 on the sense strand and -409 to -385 on the antisense strand corresponding to Fis-A4. (B) Gel shift assay of the Fis binding to the *lapA* promoter DNA containing the wild-type Fis binding site Fis-A4 and mutated site Fis-A4mut. 2 × 10^10^ molecules of radioactively labelled PCR products containing Fis-A4 and FisA4-mut sites were used for Fis binding. Fis was outcompeted from Fis-DNA complex with unlabelled PCR product containing the Fis binding site (LF2) or a PCR product without Fis binding site (RF1). Arrows point to different dissociation of Fis from radioactively labelled DNA in favour of binding unlabelled Fis-specific DNA. Added unlabelled DNA was calculated in molecules. 0.46 μM Fis was used in each reaction mixture except mixtures without Fis in lanes 2 and 12.

**Fig 6:**
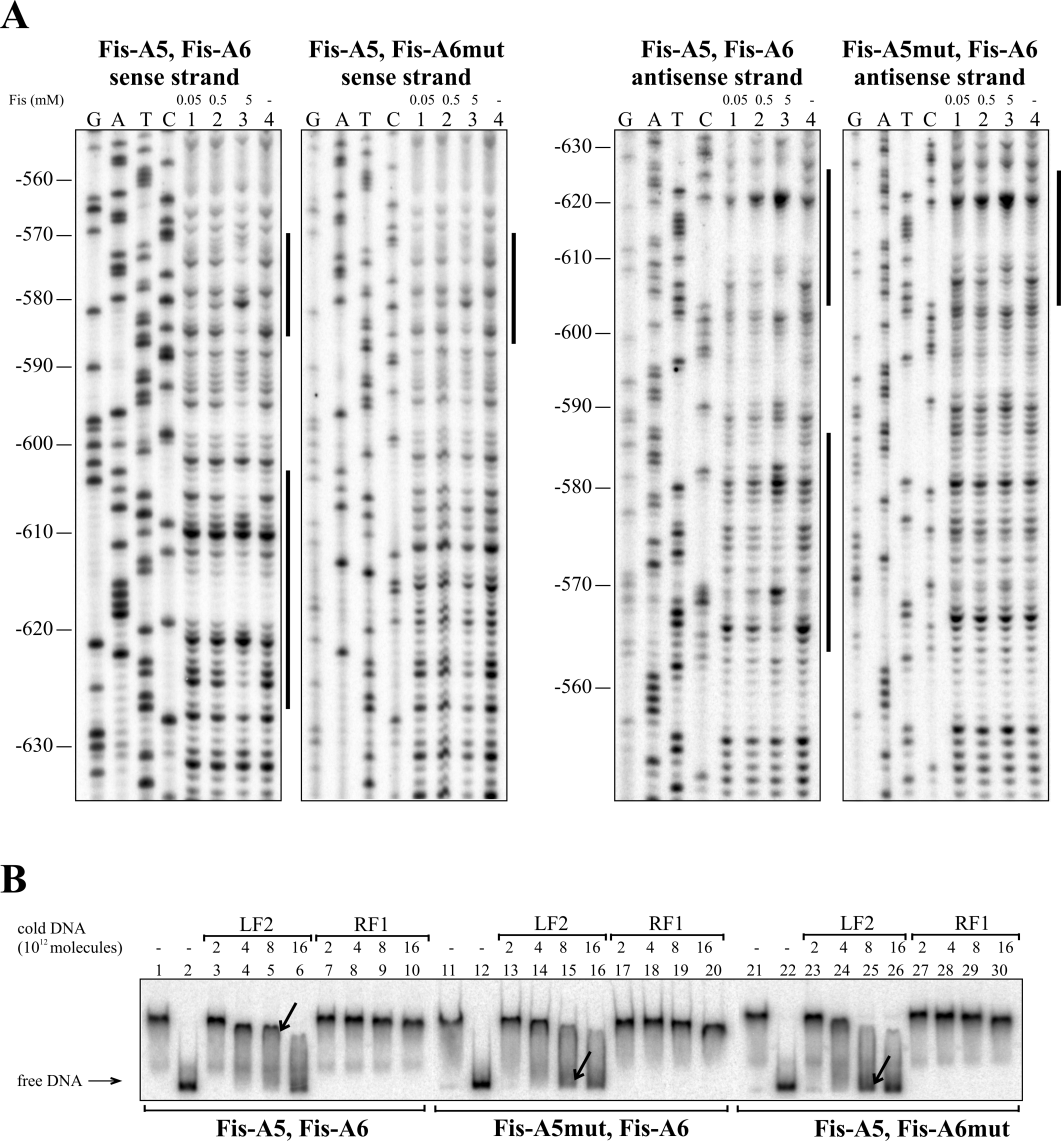
Fis binding to Fis-A5 and Fis-A6 sites upstream of the *lapA* gene. (A) Protection of the *lapA* upstream DNA against DNase I cleavage by Fis binding on the sense and the antisense strands. Lines at the right side of the panels indicate the regions protected by Fis from DNase I cleavage at the positions -586 to -566 on the sense strand and -586 to -564 on the antisense strand corresponding to Fis-A5; and -627 to -603 on the sense strand and -626 to -604 on the antisnse strand corresponding to Fis-A6. (B) Gel shift assay of the Fis binding to the *lapA* promoter DNA containing the wild-type Fis binding site Fis-A5 and Fis-A6 and one or other mutated site. 2 × 10^10^ molecules of radioactively labelled PCR products containing both Fis binding sites Fis-A5 and FisA6 or one mutated binding site FisA5mut-Fis-A6, and Fis-A5-FisA6mut were used in the assay. Fis was outcompeted from Fis-DNA complex with unlabelled PCR product containing the Fis binding site (LF2) or a PCR product without Fis binding site (RF1). Arrows point to different dissociation of Fis from radioactively labelled DNA in favour of binding unlabelled Fis-specific DNA. Added unlabelled DNA was calculated in molecules. 0.46 μM Fis was used in each reaction mixture except mixtures without Fis in lanes 2, 12 and 22.

**Fig 7:**
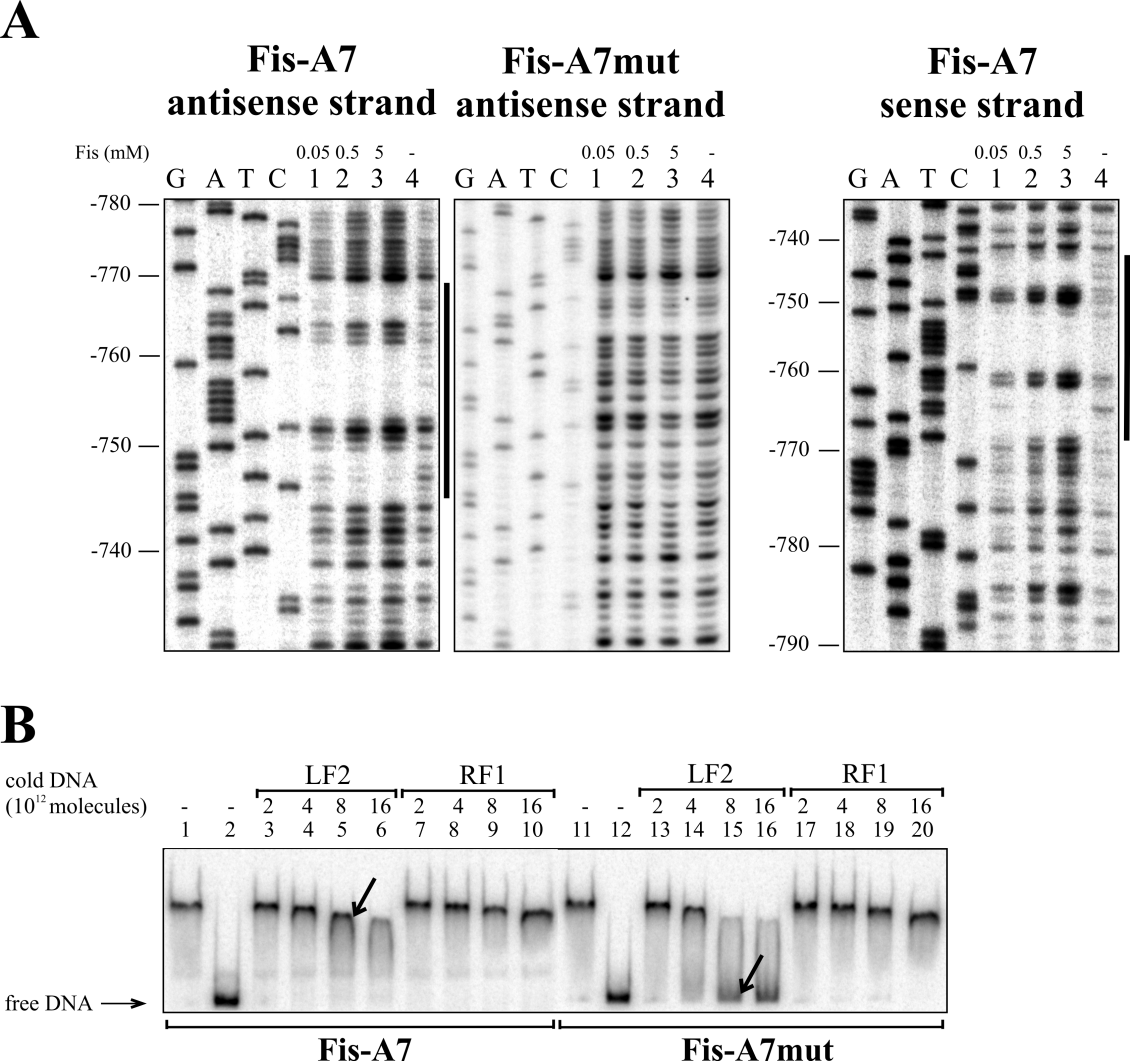
Fis binding to Fis-A7 site upstream of the *lapA* gene. (A) Protection of the *lapA* upstream DNA against DNase I cleavage by Fis binding on the sense and the antisense strands. Lines at the right side of the panels indicate the regions protected by Fis from DNase I cleavage at the positions -744 to -768 on the sense strand and -745 to -769 on the antisense strand corresponding to Fis-A7. (B) Gel shift assay of the Fis binding to the *lapA* promoter DNA containing the wild-type Fis binding site Fis-A7 and mutated site Fis-A7mut. 2 × 10^10^ molecules of radioactively labelled PCR products containing Fis-A7 and Fis-A7mut sites were used for Fis binding. Fis was outcompeted from Fis-DNA complex with unlabelled PCR product containing the Fis binding site (LF2) or a PCR product without Fis binding site (RF1). Arrows point to different dissociation of Fis from radioactively labelled DNA in favour of binding unlabelled Fis-specific DNA. Added unlabelled DNA was calculated in molecules. 0.46 μM Fis was used in each reaction mixture except mixtures without Fis in lanes 2 and 12.

Eight Fis binding sequences, Fis-A1 to Fis-A8, were predicted for the *lapA* promoter region *in silico* (Table 2). DNase I footprint analysis verified six of the Fis binding sites: Fis-A1 at approximately -95 bp relative to the *lapA* gene, Fis-A2 at -145 bp (Figs 2A and 4A); Fis-A4 at -400 bp (Figs 2A and 5A) Fis-A5 at -575 bp, Fis-A6 at -615 bp (Figs 2A and 6A) and Fis-A7 at -755 bp relative to the *lapA* gene (Figs 2A and 7A). We could not verify Fis binding to the predicted Fis-A3 and Fis-A8 sequences by DNase I footprint analysis (data not shown).

**Table 2:** Sequences of *in silico* predicted Fis binding sites in the upstream region of *lapA* gene.

Name of site	Sequence	Strand	Positions from start codon	Weight score^a^	P-value
Fis-A1	GACGTCAATATTCCGTCTAT	Antisense	-117…-98	5.7	4.7 × 10^−4^
Fis-A2	AAGGTCAATAGTTTGGCAGT	Sense	-156…-137	7.5	6.4 × 10^−5^
	CTGCCAAACTATTGACCTTA	Antisense	-157…-138	6.5	2.0 × 10^−4^
Fis-A3	ATGTAAGACATTTGTCCTCA	Sense	-245…-226	5.7	4.7 × 10^−4^
Fis-A4	GTGCTTATTTGGCAATCAGT	Sense	-404…-385	5.4	6.3× 10^−4^
	CTGATTGCCAAATAAGCACA	Antisense	-405…-386	5.6	5.1 × 10^−4^
Fis-A5	TTGCATTCAATATCCGCAAG	Sense	-584…-565	5.5	5.7 × 10^−4^
	TTGCGGATATTGAATGCAAG	Antisense	-585…-566	5.4	6.3 × 10^−4^
Fis-A6	GTTAGTCAAAATTCATCTAT	Sense	-625…-606	6.6	1.8 × 10^−4^
	TAGATGAATTTTGACTAACA	Antiense	-626…-607	5.6	5.1 × 10^−4^
Fis-A7	TTGTTTCATTTTTGATCCAG	Sense	-765…-746	7.3	8.1 × 10^−5^
	TGGATCAAAAATGAAACAAT	Antisense	-766…-747	7.4	7.2 × 10^−5^
Fis-A8	ACGTAGAATTGTTTACTCAA	Antisense	-818…-799	5.3	6.9 × 10^−4^

^*a*^ The maximum possible weight score for the Fis matrix was 12.5.

Thereafter, all verified Fis binding sites were mutated considering the most important nucleotides in *E*. *coli* Fis binding sites [46,47]. Enough positions were chosen for nucleotide substitutions to avert *in silico* binding [43] to the sites. Mutated Fis binding sites (Fig 2A) were used to confirm direct Fis binding to the *lapA* promoter region. The DNase I footprint analysis revealed that unlike the wild-type Fis binding sites, Fis did not prevent DNase I cleavage of the Fis-A1mut, A2mut, A5mut, A6mut or A7mut sequences (Figs 4A, 6A and 7A). Fis-A4 was an exception because it required more substitutions in the Fis binding site than were predicted *in silico*. First, five substitutions were made in Fis-A4 that averted *in silico* binding of Fis, but maintained the functionality of the promoter P_*lapA6*_. However, the five substitutions in the P_*lapA6*_*−*10 box flanking sequence did not hinder Fis protection of Fis-A4 against DNase I cleavage (data not shown). More extensive mutation of Fis-A4 that included substitutions in the -10 box of P_*lapA6*_ (Fig 2A) abolished Fis-A4mut protection by Fis against DNase I (Fig 5A).

Additionally, Fis binding to the six determined binding sequences was assessed by gel mobility shift analysis. To assess Fis-specific binding, unlabelled DNA containing the LF2 Fis binding site from the left end of Tn*4652* [44] was used to outcompete Fis from the *lapA* promoter DNA-Fis complex (Figs 4B, 5B, 6B and 7B). Although Fis bound relatively similarly to the wild-type and mutated DNA fragments, LF2 outcompeted Fis from all of the complexes with mutated DNA fragments more easily than those with wild-type Fis binding sites (Figs 4B, 5B, 6B and 7B). Mutating Fis-A4 (Fig 5B, compare lanes 5 and 15) and Fis-A7 (Fig 7B, compare lanes 5 and 15) enabled out competition by LF2 the most. Mutating Fis-A1, Fis-A2, Fis-A5 or Fis-A6 had smaller effects. This can be due to the presence of Fis-A1 and Fis-A2 binding sites within the same DNA fragment. Mutating one site still left the other one unaffected and able to bind Fis, thereby hindering the out-competition by LF2. The same applied to Fis-A5 or Fis-A6, which are also together in one PCR fragment.

Altogether, DNase I footprint and gel mobility shift verified Fis binding to six binding sites (Fig 2) upstream of the *lapA* gene *in vitro*. Mutating Fis binding sites averted Fis binding and enabled easier outcompetition by Fis-specific DNA.

### The positive effect of Fis on *lapA* transcription depends on Fis-A5 and Fis-A7

**Fig 8:**
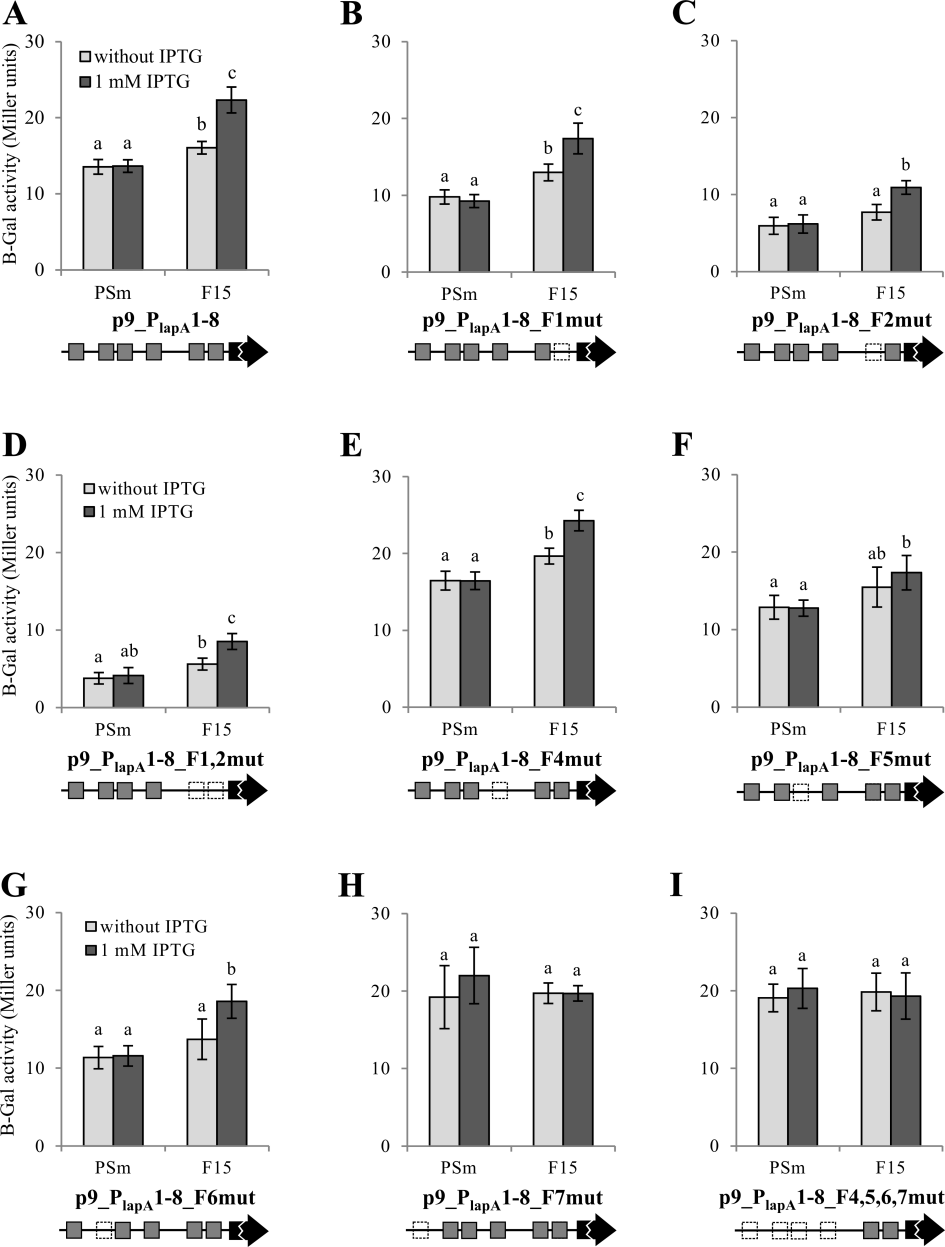
The effect of mutated Fis binding sites in the *lapA* promoter region on the level of reporter gene *lacZ* expression. B-galactosidase (β-Gal) activity expressed from the *lapA* promoter-*lacZ* reporter constructs was measured in *P*. *putida* wild-type strain PSm and *fis* overexpression strain F15 grown in LB medium with or without 1 mM IPTG for 18 hours. Schemes of Fis binding sites (shown as grey boxes) are shown below the diagrams. Dotted lines denote mutated Fis binding sites and the *lacZ* reporter gene is shown as a black arrow. The scheme is not to scale. Vertical bars denote 95% confidence intervals of means. Data of at least 5 independent measurements is shown. Letters a–c depict homogeneity groups according to ANOVA *post hoc* Bonferroni test. Within subfigures, identical letters denote non-significant differences (*P*>0.05) between averages of β-galactosidase activity.

To investigate the possible effect of Fis on the transcription of the *lapA* gene, the 951 bp DNA fragment containing all of the investigated potential *lapA* promoters was cloned in front of the *lacZ* reporter gene in the low-copy-number promoter probe vector p9TT_B_lacZ (S1 Table). B-galactosidase activity was measured in stationary-phase PSm (wild-type) and F15 (IPTG-inducible *fis* overexpression strain, [20]) cells (Fig 8 and S3 Table). The *fis* overexpression strain was used as *fis* deletion is lethal to *Pseudomonas* species [20,31,53] and conditional expression strains were unstable (data not shown).

**Fig 9:**
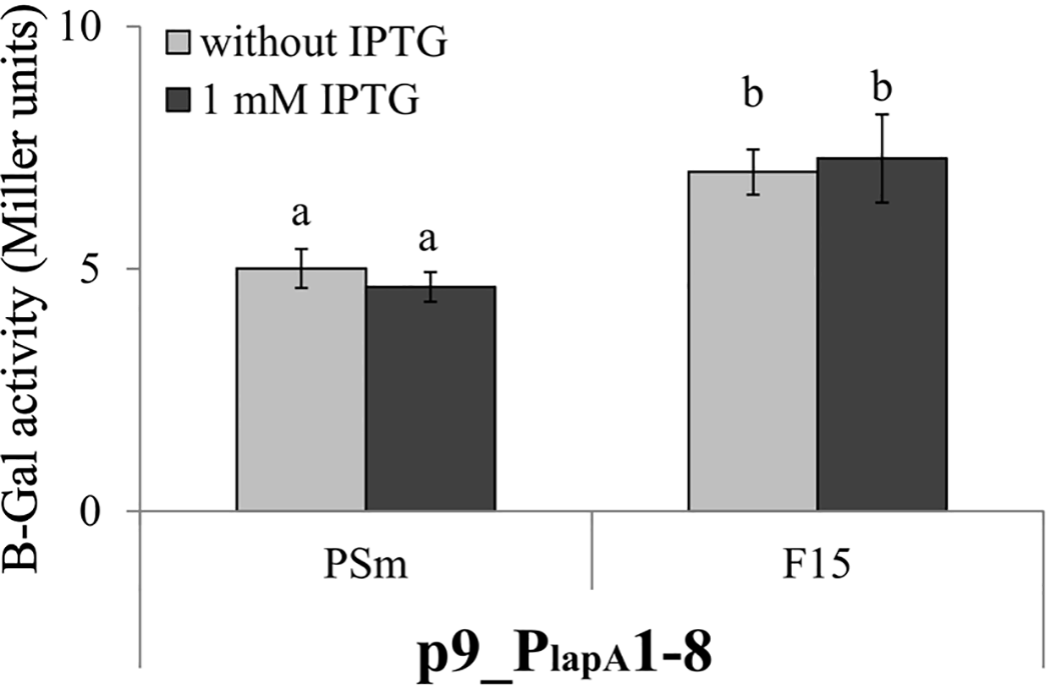
The effect of *fis* overexpression to the transcription of *lapA* promoter region in exponential phase *P*. *putida*. Β-galactosidase (β-Gal) activity expressed from the *lapA* promoter-*lacZ* reporter constructs was measured in *P*. *putida* wild-type strain PSm and *fis* overexpression strain F15 grown in LB medium with or without 1 mM IPTG to the optical density of 0.5. Vertical bars denote 95% confidence intervals of means. Data of at least 10 independent measurements is shown. Letters a–b depict homogeneity groups according to ANOVA *post hoc* Bonferroni test. Identical letters denote non-significant differences (*P*>0.05) between averages of β-galactosidase activity.

The *P*. *putida* strains PSm and F15 harbouring promoterless p9TT_B_lacZ showed a β-galactosidase activity of 0.21 to 0.45 Miller units (S3 Table). The β-galactosidase activity of PSm did not depend on added IPTG as the investigated vectors ensured a similar LacZ activity in cells grown with or without IPTG (Figs 8 and 9). This demonstrated that IPTG itself has no effect on the expression of the *lacZ* reporter gene. IPTG-induced *fis* overexpression in the F15 strain harbouring an additional *fis* gene copy under the P_*tac*_ promoter [20] increased the activity of the *lapA* promoter region 1.4 times compared to no IPTG supplementation, indicating that Fis activates the transcription of *lapA* in stationary phase (Fig 8A). No effect of *fis* overexpression was observed in exponentially growing *P*. *putida* (Fig 9). This result was expected because we have seen the impact of *fis* overexpression on the amount of LapA in stationary phase cells and on biofilm after 24 hours of inoculation but not in exponentially growing bacteria or 4-hours-old biofilm of *P*. *putida* [4].

To investigate which Fis binding sites are used for the activation of *lapA* transcription in the stationary phase, we used mutated Fis binding sites. Substitutions in the sites Fis-A1 and/or Fis-A2 did not abolish the positive effect of Fis-overexpression on the activity of LacZ (Fig 8B–8D). Therefore, these sites alone in the *lapA* promoter area did not ensure the Fis’ positive effect on transcription. Constructs p9_P_lapA_1-8_F4mut and p9_P_lapA_1-8_F6mut also displayed Fis-induced activation (Fig 8E and 8G), showing that Fis-A4 and Fis-A6 are not individually responsible for the Fis-induced transcription activation of *lapA*. However, Fis-A5 and Fis-A7 seem to be important for the Fis induced *lapA* expression as mutating either one diminished the Fis-induced increase in β-galactosidase activity (Fig 8F and 8H) characteristic to the native *lapA* promoter region. As a control, we also measured the β-galactosidase activity of the construct p9_P_lapA_1-8_F4,5,6,7mut with all distal binding sites mutated together and unsurprisingly it also had no Fis-induction effect (Fig 8I).

### Fis has a direct effect only to P_*lapA8*_ among the distal individual promoters

We were interested in Fis’ influence on transcription of three promoters P_*lapA6*_, P_*lapA7*_ and P_*lapA8*_ as Fis binding sites from Fis-A4 to Fis-A7 were located in positions that could influence the transcription from these promoters directly. Near the promoter P_*lapA6*_ is one Fis binding site, Fis-A4, which overlaps the -10 box of the promoter (Fig 2). Regulator binding sites in such positions generally repress transcription. The Fis binding site Fis-A5 is located upstream of the promoter P_*lapA7*_ (Fig 2). There are two Fis binding sites near P_*lapA8*_: Fis-A6 overlaps the -10 box of P_*lapA8*_ and Fis-A7 is located upstream of the promoter in position -155 from the 5ʹ end A-VIII (Fig 2).

**Fig 10:**
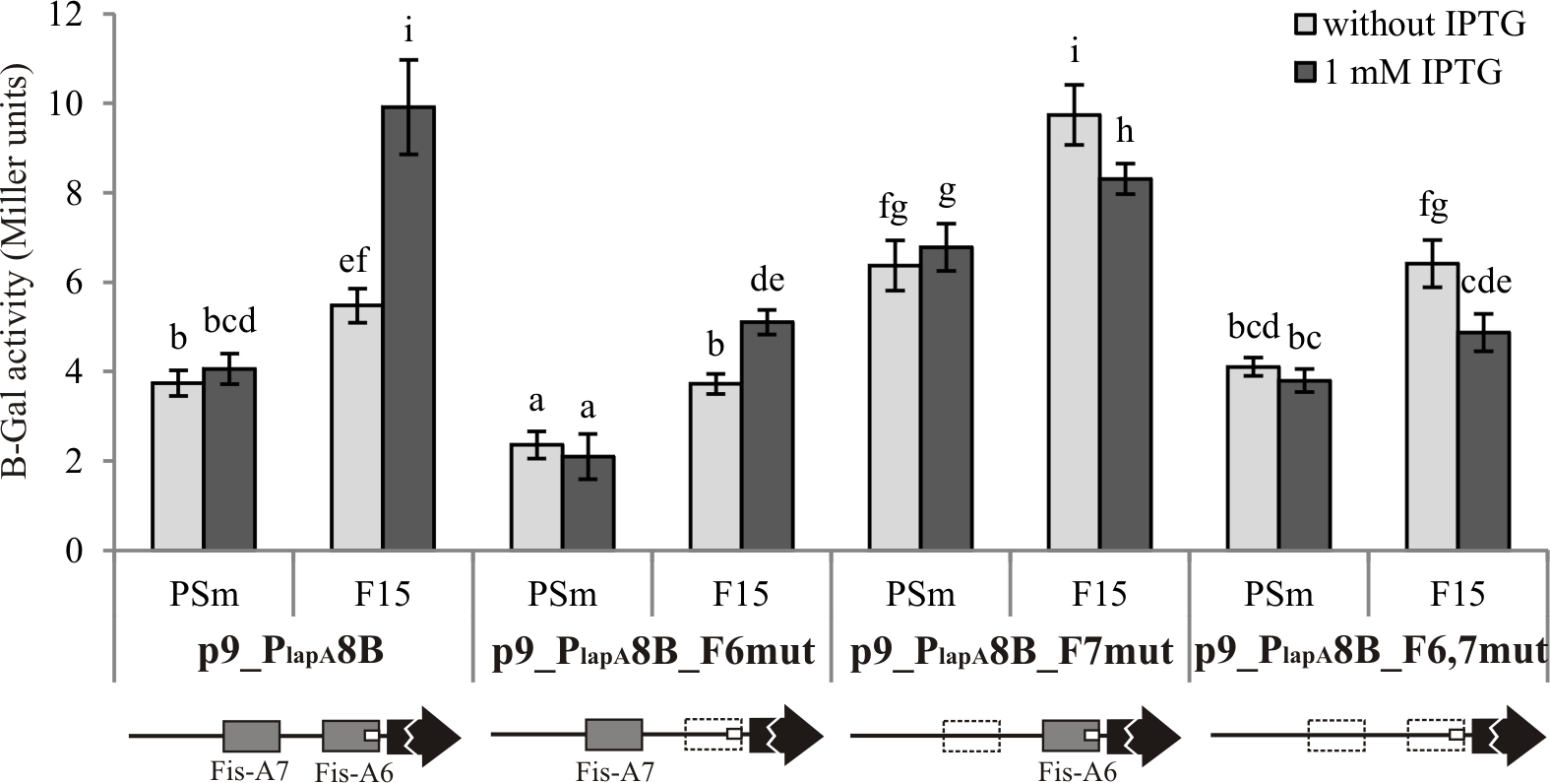
The effect of mutated Fis binding sites Fis-A6 and Fis-A7 to P_*lapA8*_ promoter. B-galactosidase (β-Gal) activity expressed from the *lapA* promoter-*lacZ* reporter constructs p9_P_lapA_8B, p9_P_lapA_8B_F6mut, p9_P_lapA_8B_F7mut and p9_P_lapA_8B_F6,7mut were measured in *P*. *putida* wild-type strain PSm and *fis* overexpression strain F15 grown in LB medium with or without 1 mM IPTG for 18 hours. Schemes of Fis binding sites (shown as grey boxes) are shown below the diagrams. Dotted lines denote mutated Fis binding sites, *lacZ* reporter gene is shown as a black arrow and promoter P_*lapA8*_ in a small white box. The scheme is not to scale. Vertical bars denote 95% confidence intervals of means. Data of at least 9 independent measurements is shown. Letters a–i depict homogeneity groups according to ANOVA *post hoc* Bonferroni test. Identical letters denote non-significant differences (*P*>0.05) between averages of β-galactosidase activity.

Fis-A7 is necessary for the Fis enhanced transcription activation from the most distal *lapA* promoter—P_*lapA8*_. *Fis* overexpression induced by IPTG in F15 increased the activity of P_*lapA8*_ 1.8 times compared to no IPTG supplementation. Mutating the Fis-A7 binding site abolished the positive effect of *fis* overexpression on the activity of P_*lapA8*_ (Fig 10). Mutating the Fis-A6 binding site overlapping the same promoter did decrease the overall transcription, but the positive effect of *fis* overexpression was still present (Fig 10). This indicated that Fis does not affect P_*lapA8*_ via binding to Fis-A6.

**Fig 11:**
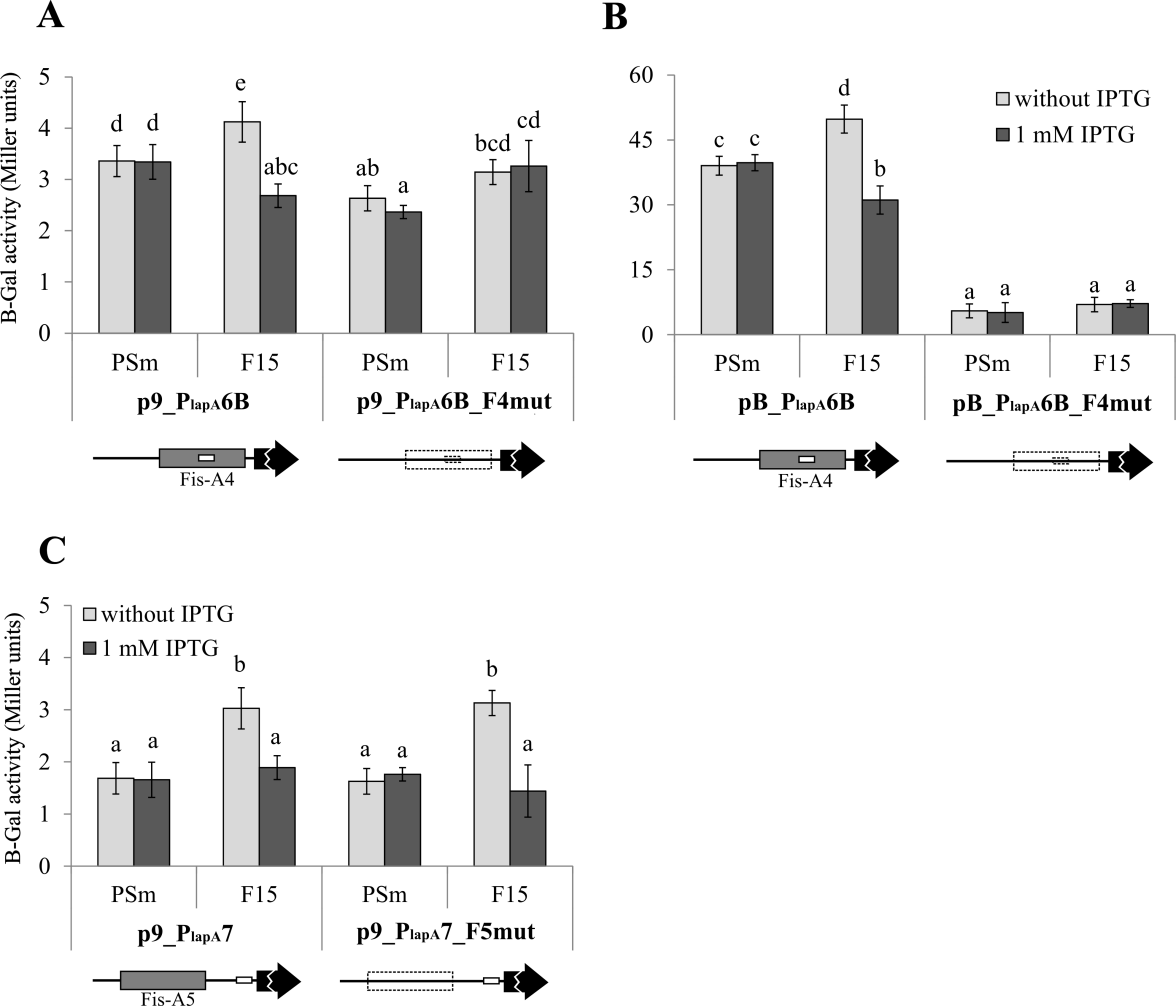
The effect of mutated Fis binding sites to individual promoters P_*lapA6*_ and P_*lapA7*_. B-galactosidase (β-Gal) activity expressed from the *lapA* promoter-*lacZ* reporter constructs. (A) P_*lapA6*_ promoter construct with or without mutated Fis-A4 binding site was cloned into medium-copy plasmid pBLKT and low-copy plasmid p9TT_B_lacZ. p9_P_lapA_6B, p9_P_lapA_6B_F4mut, (B) pB_P_lapA_6B, pB_P_lapA_6B_F4mut, (C) p9_P_lapA_7 and p9_P_lapA_7_F5mut were measured in *P*. *putida* wild-type strain PSm and *fis* overexpression strain F15 grown in LB medium with or without 1 mM IPTG for 18 hours. Schemes of Fis binding sites (shown as grey boxes) are shown below the diagrams. Dotted lines denote mutated Fis binding sites and mutated promoter P_*lapA6*_, *lacZ* reporter gene is shown as a black arrow and promoters P_*lapA6*_ and P_*lapA7*_ as white boxes. The scheme is not to scale. Vertical bars denote 95% confidence intervals of means. Data of at least 9 independent measurements is shown. Letters a–e depict homogeneity groups according to ANOVA *post hoc* Bonferroni test. Within subfigures, identical letters denote non-significant differences (*P*>0.05) between averages of β-galactosidase activity.

*Fis* overexpression seemed to repress the activity of the individual promoter P_*lapA7*_ (Fig 11C), but mutations in Fis-A5 had no effect on the P_*lapA7*_ activity. However, in the above-described experiment with the 951 bp long *lapA* promoter area, we observed the positive effect of *fis* overexpression on *lapA* transcription, which depended on the functionality of the Fis-A5 binding site (Fig 8). Thus, Fis is likely still able to weakly bind the mutated Fis-binding site Fis-A5mut *in vivo*. The binding may be sufficient to repress the transcription from P_*lapA7*_, but not to activate the transcription of *lapA* via DNA topology modification. Considering the fact that P_*lapA7*_ is a weak promoter (Table 1 and Fig 11C), Fis binding to Fis-A5 affects *lapA* expression mostly by modifying *lapA* upstream DNA topology rather than regulating transcription from the individual promoter P_*lapA7*_.

The impact of Fis-A4 binding site to the transcriptional activity of P_*lapA6*_ promoter remained unclear. To assess the impact of Fis to the transcription from P_*lapA6*_, we cloned the whole Fis binding site Fis-A4 mapped by DNAse I footprint analysis into p9TT_B_lacZ, resulting in p9_P_lapA_6B (S1 Table). This construct contains 28 extra nucleotides of the *lapA* promoter area in the 3ʹ end compared to pB_P_lapA_6 (Fig 1). Binding of Fis to Fis-A4 repressed the activity of P_*lapA6*_, but in a moderate manner, 1.53 times (Fig 11A and 11B). Mutating Fis-A4 also disrupted the P_*lapA6*_ promoter as we were unable to hinder Fis binding without damaging the -10 box (data not shown). Therefore, we were unable to distinguish the effects of mutating Fis binding sites from the effects of mutating the promoter.

The loss of transcription from the promoter P_*lapA6*_ with mutated -10 box depended on the copy number of the vector. Plasmid pB_P_lapA_6mut used in the promoter verification experiment (Table 1) and p9_P_lapA_6B_F4mut used to study the impact of Fis binding to the site Fis-A4 (Fig 11A) both contained identically mutated -10 boxes of P_*lapA6*_ promoter but the extent of negative effect of mutated -10 boxes differed in these constructs. Compared to the wild-type variant, the LacZ activites were 1.28 and 14.33 times lower in PSm harbouring the respective high and low copy number constructs with mutated P_*lapA6*_ (Table 1, Fig 11A and 11B). Compared to pB_P_lapA_6mut, the construct p9_P_lapA_6B_F4mut had 5 additional substitutions in the DNA flanking the -10 box due to mutation in the Fis-A4 binding site (Fig 2). To assess whether the mutation of Fis-A4 binding-site present in plasmid p9_P_lapA_6B_F4mut could affect the transcription from P_*lapA6*_, the DNA fragments contaning P_lapA_6B and P_lapA_6B_F4mut sequences were cloned to the medium-copy-number plasmid pBLKT, resulting in pB_P_lapA_6B and pB_P_lapA_6B_F4mut (S1 Table). Indeed, the loss of transcription from the P_*lapA6*_ in the medium-copy-number plasmid pBLKT was independent from the number of substitutions in promoter area (Table 1 and Fig 11B).

Altogether, our results indicate that the positive effect of *fis* overexpression on the transcription of the *lapA* gene depends on the Fis binding sites Fis-A5 and Fis-A7, where Fis-A7 influences the transcription from the P_*lapA8*_ promoter and Fis-A5 is probably involved in transcriptional regulation of *lapA* by modification of DNA topology of whole promoter area.

## Discussion

LapA is the largest protein of *P*. *putida* and it is a key factor for biofilm formation in this bacterium [4,11]. LapA’s posttranslational regulation is well described but relatively little is known about the transcriptional regulation. As the excact number and location of *lapA* promoters was unknown, our first aim was to identify the promoters of *lapA*. We determined six σ^70^ type (RpoD) promoters upstream of *lapA* by RACE and by mutating potential -10 boxes of these promoters (Table 1, Figs 1 and 2). All the identified promoters of *lapA* were negatively affected by the substitutions in potential -10 boxes (Table 1) and were active in exponentially growing bacteria as well as in stationary phase cells. Thus, the inability to identify the 5ʹ ends of *lapA* mRNA corresponding to promoters P_*lapA5*_ and P_*lapA8*_ in exponential phase may have been due to technical reasons. However six promoters is an unusually high number, as most tested *E*. *coli* genes are proposed to have one or two promoters [54,55] indicating the complexity of transcriptional regulation. Indeed, some of the promoters (for example P_*lapA6*_ and P_*lapA7*_) provide only low transcription in LB-grown *P*. *putida* (Table 1) and thereby the contribution of these promoters to the expression of *lapA* seems insignificant. Nonetheless, the activity of promoters can depend on factors found in specific environments that may be absent in classically used growth media. Therefore, in laboratory conditions the transcription from such promoters may be downregulated. For instance, the *algD* transcription in *P*. *putida* KT2440 is strongly activated in the rhizosphere of maize roots and undetectable or at basal level in M9-citrate medium [56]. *AlgD* is the first gene in the *algD-8-44KEGXLIJFA* operon, which is responsible for the biosynthesis of alginate, the exopolysaccharide that is an important component of mucoid *P*. *aeruginosa* biofilm [57,58]. As the substitutions in the potential -10 boxes of P_*lapA6*_ and P_*lapA7*_ decreased LacZ activity 8 to 20 times (Table 1), it seems that P_*lapA6*_ and P_*lapA7*_ are essentially functional promoters that are weak in LB medium, yet maybe differently regulated in the nature.

Out of the identified promoters, P_*lapA3*_ seems to be the most important and the most proximal promoter for *lapA* transcription in LB medium (Fig 2A, Table 1). LacZ activities measured in LB-grown *P*. *putida* harbouring pB_P_lapA_1 or pB_P_lapA_1-2, which respectively contained one or two most proximal hypothetical *lapA* promoters, were insignificant (Table 1). Similarly to the previous two plasmids, insignificant LacZ activity was measured in LB-grown *P*. *putida* wild-type strain PSm carrying pB_P_lapA_1-3_P_lapA_3mut, which carries a mutated P_*lapA3*_ promoter.

Our results indicate that the stationary phase sigma factor RpoS can be involved in the regulation of the three distal *lapA* promoters, P_*lapA6*_, P_*lapA7*_ and P_*lapA8*_ (Table 1). The effects of *rpoS* deletion were moderate, indicating partial σ^S^-dependence of these promoters. As RpoS and RpoD can recognize a similar promoter consensus [59,60] these promoters are probably recognized by both sigma factors, RpoD and RpoS.

The transcription regulation of *lapA* seems to be under the control of many regulators. In search for the *lapA* promoters, we extended the upstream region of *lapA* in the promoter probe vector, adding one hypothetical promoter at a time and expected to see an increase in activity every time a promoter is added (Table 1 and Fig 1). However, adding potential promoters to the construct did not always increase the activity of the promoter construct and adding one particular promoter P_*lapA7*_ even decreased the activity. This indicates that the extension of upstream DNA in front of the reporter gene has not added only promoters, but other regulatory areas as well.

So far, only activators have been described for *lapA*: FleQ, c-di-GMP, GacS and Fis have been shown to regulate *lapA* expression [4,16–18]. Indeed, we have previously shown that overexpressing the global transcription regulator Fis increases the amount of the LapA protein 1.6 times compared to the wild-type cells [4]. Our current work elucidates the mechanisms by which Fis activates *lapA* expression. We observed that *fis* overexpression increases the transcription of *lapA* 1.4 times (Fig 8A). Fis binds to the *lapA* promoter region in six specific sites *in vitro* and mutating these sites hinders binding (Figs 4–7). Fis’ positive effect on *lapA* transcription *in vivo* depends on the two distal Fis binding sites, Fis-A5 and Fis-A7 (Fig 8A, 8F and 8H). The rest of the Fis binding sites: Fis-A1, Fis-A2, Fis-A4 and Fis-A6 have a redundant impact, if any, to *lapA* transcription. However, the importance of the Fis-A4 binding site to the regulation of *lapA* transcription stays unclear, as we were unable to separate the effects of mutating the Fis-A4 binding site and the P_*lapA6*_ promoter that overlaps it (Table 1 and Fig 11A). Two mechanisms could explain the positive effect of Fis on the transcription of *lapA*. Firstly, Fis can enhance *lapA* transcription only from one individual promoter, P_*lapA8*_, which expression depends on Fis binding to Fis-A7 (Fig 10). Secondly, Fis can regulate *lapA* transcription by modifying the topology of upstream DNA. Indeed, the Fis binding site Fis-A5 is not important for the transcriptional activation from its nearest promoter P_*lapA7*_ (Fig 11C) but it affects transcription in the presence of all promoters (Fig 8F). Also, we cannot exclude the possibility that Fis-A1, Fis-A2, Fis-A4 and/or Fis-A6 binding sites could contribute to the *lapA* promoter area topology. It is likely that the two mechanisms work synergistically: Fis activates transcription of the P_*lapA8*_ promoter directly and also changes the DNA topology of the whole *lapA* promoter area.

Mutating potential Fis binding sites Fis-A1 and/or Fis-A2 does not abolish *fis* overexpression’s activating effect on *lapA* transcription but the overall activity of the *lapA* 951-bp-long promoter region decreases (Fig 8), indicating the importance of this region. Jimenez-Fernandez and other have *in silico* predicted three FleQ binding sites positioned at nucleotides -101 to -114, -140 to -153 and -655 to -668 [18]. Surprisingly, two of the proximal sites overlap with Fis binding sites Fis-A1 and Fis-A2 (Fig 2). The third predicted FleQ binding site is located between sites Fis-A6 and Fis-A7 (Fig 2). However, the exact FleQ binding positions in *lapA* upstream DNA have not been specified because FleQ binding has only been shown by gel mobility shift [18]. Mutating the Fis-A2 site also substituted three nucleotides in the predicted overlapping FleQ binding site and mutating the Fis-A1 site changed 6 nucleotides adjacent to the predicted FleQ binding site. This means these substitutions may disrupt FleQ binding and thereby decrease the total level of transcription from *lapA* promoter region.

This raises the question, how could FleQ binding to these two sites regulate *lapA* transcription in LB-grown *P*. *putida* when these sites are located downstream of all the proven promoters active in LB-grown bacteria. Activators binding downstream of promoters are uncommon in bacteria. However, FleQ seems to be an exception as *Pseudomonas aeruginosa* FleQ can activate transcription of *flhA* by binding downstream of the promoter [61]. Therefore, it is possible that FleQ activates *lapA* via binding sites located downstream of the promoters. The third *in silico* predicted FleQ binding site is located between P_*lapA8*_ and the Fis binding site Fis-A7. It is possible that Fis and FleQ regulate *lapA* transcription from P_*lapA8*_ promoter co-operatively. However, as the exact binding sites for FleQ remain unknown, it is impossible to propose an exact mechanism.

Biofilm development and *fis* expression follow similar trends in LB-grown *P*. *putida*. Both are up-regulated in a nutrient-rich environment and down-regulated or hindered by nutrient depletion. We have shown that *P*. *putida* biofilm is the strongest after 4 hours of inoculation in LB medium and it decays about threefold within the next 4 hours [4]. While exact numbers vary, others have described similar trends [5–7]. Fis mRNA levels are highest in exponentially growing planktonic *P*. *putida* and drop approximately three times in stationary phase cells [19]. It is not surprising that *fis* overexpression activates *lapA* transcription (Fig 8A) and increases the amount of LapA protein [4] resulting in enhanced mature biofilm [4,20]. At the same time, *fis* overexpression does not affect the 4-hours-old biofilm [4] or the expression of *lapA* in exponentially growing *P*. *putida* (Fig 9) [4]. Considering the increased expression of *fis* in exponentially growing wild-type cells [19], the native amount of Fis in fast growing *P*. *putida* could be enough to saturate the Fis binding sites Fis-A5 and Fis-A7 and enhance *lapA* expression. As the native *fis* expression decreases in stationary phase, artificial overexpression of Fis allows the detectable positive regulation of *lapA*.

## Conclusion

Six promoters and Fis are involved in the complex regulation of *lapA* transcription in LB medium. Two of the six Fis binding sites are necessary for the Fis-enhanced expression of *lapA in vivo*. Fis activates the transcription of *lapA* from the most distal promoter P_*lapA8*_ and can additionally regulate *lapA* transcription via modification of *lapA* upstream DNA topology.

## Supporting information

S1 Table Bacterial strains and plasmids used in this study. (DOCX)

S2 Table Oligonucleotides used in this study. ^*a*^ restrictases are shown in brackets. ^*b*^ sequences recognized by endonucleases are underlined, the nucleotides mutated in Fis binding sites or -10 boxes are shown in bold and sequences complementary to another oligonucleotide are indicated in bold and italics.
(DOCX)

S3 Table B-galactosidase activity (Miller units) in *P. putida* strains PSm and F15 harbouring promoterless p9TTBlacZ. Data from at least 5 independent measurements is shown. 95% confidence intervals are shown in parentheses. Letters a-c depict different homogeneity groups according to ANOVA *post hoc* Bonferroni test. Identical letters denote non-significant differences (*P*>0.05) between averages of β-galactosidase activity.
(DOCX)

## References

[1] Wolska KI, Grudniak AM, Rudnicka Z, Markowska K (2015) Genetic control of bacterial biofilms. J Appl Genet. 10.1007/s13353-015-0309-2 PMC4830867 26294280

[2] Fazli M, Almblad H, Rybtke ML, Givskov M, Eberl L, Tolker-Nielsen T (2014) Regulation of biofilm formation in *Pseudomonas* and *Burkholderia* species. Environ Microbiol 16: 1961–1981. doi: 10.1111/1462-2920.12448 24592823 10.1111/1462-2920.12448

[3] Waite RD, Paccanaro A, Papakonstantinopoulou A, Hurst JM, Saqi M, Littler E, et al (2006) Clustering of *Pseudomonas aeruginosa* transcriptomes from planktonic cultures, developing and mature biofilms reveals distinct expression profiles. BMC Genomics 7: 162 doi: 10.1186/1471-2164-7-162 16800888 10.1186/1471-2164-7-162 PMC1525188

[4] Moor H, Teppo A, Lahesaare A, Kivisaar M, Teras R (2014) Fis overexpression enhances *Pseudomonas putida* biofilm formation by regulating the ratio of LapA and LapF. Microbiology 160: 2681–2693. doi: 10.1099/mic.0.082503-0 25253613 10.1099/mic.0.082503-0

[5] Yousef-Coronado F, Travieso ML, Espinosa-Urgel M (2008) Different, overlapping mechanisms for colonization of abiotic and plant surfaces by *Pseudomonas putida*. FEMS Microbiol Lett 288: 118–124. doi: 10.1111/j.1574-6968.2008.01339.x 18783437 10.1111/j.1574-6968.2008.01339.x

[6] Yousef-Coronado F, Soriano MI, Yang L, Molin S, Espinosa-Urgel M (2011) Selection of hyperadherent mutants in *Pseudomonas putida* biofilms. Microbiology-Sgm 157: 2257–2265. 10.1099/mic.0.047787-0 21602214

[7] Gjermansen M, Nilsson M, Yang L, Tolker-Nielsen T (2010) Characterization of starvation-induced dispersion in *Pseudomonas putida* biofilms: genetic elements and molecular mechanisms. Mol Microbiol 75: 815–826. doi: 10.1111/j.1365-2958.2009.06793.x 19602146 10.1111/j.1365-2958.2009.06793.x

[8] Duque E, de la Torre J, Bernal P, Molina-Henares MA, Alaminos M, Espinosa-Urgel M, et al (2013) Identification of reciprocal adhesion genes in pathogenic and non-pathogenic *Pseudomonas*. Environ Microbiol 15: 36–48. doi: 10.1111/j.1462-2920.2012.02732.x 22458445 10.1111/j.1462-2920.2012.02732.x

[9] Martinez-Gil M, Yousef-Coronado F, Espinosa-Urgel M (2010) LapF, the second largest *Pseudomonas putida* protein, contributes to plant root colonization and determines biofilm architecture. Mol Microbiol 77: 549–561. doi: 10.1111/j.1365-2958.2010.07249.x 20545856 10.1111/j.1365-2958.2010.07249.x

[10] El-Kirat-Chatel S, Beaussart A, Boyd CD, O'Toole GA, Dufrene YF (2014) Single-cell and single-molecule analysis deciphers the localization, adhesion, and mechanics of the biofilm adhesin LapA. ACS Chem Biol 9: 485–494. doi: 10.1021/cb400794e 24556201 10.1021/cb400794e PMC4930830

[11] Espinosa-Urgel M, Salido A, Ramos JL (2000) Genetic analysis of functions involved in adhesion of *Pseudomonas putida* to seeds. J Bacteriol 182: 2363–2369. 10762233 10.1128/jb.182.9.2363-2369.2000 PMC111295

[12] Hinsa SM, Espinosa-Urgel M, Ramos JL, O'Toole GA (2003) Transition from reversible to irreversible attachment during biofilm formation by *Pseudomonas fluorescens* WCS365 requires an ABC transporter and a large secreted protein. Mol Microbiol 49: 905–918. 12890017 10.1046/j.1365-2958.2003.03615.x

[13] Lahesaare A, Ainelo H, Teppo A, Kivisaar M, Heipieper HJ, Teras R (2016) LapF and its regulation by Fis affect the cell surface hydrophobicity of *Pseudomonas putida*. PLoS One 11: e0166078 doi: 10.1371/journal.pone.0166078 27812186 10.1371/journal.pone.0166078 PMC5094663

[14] Navarro MVAS, Newell PD, Krasteva PV, Chatterjee D, Madden DR, O'Toole GA, et al (2011) Structural basis for c-di-GMP-mediated inside-out signaling controlling periplasmic proteolysis. Plos Biology 9. 10.1371/journal.pbio.1000588 PMC3032553 21304926

[15] Newell PD, Boyd CD, Sondermann H, O'Toole GA (2011) A c-di-GMP effector system controls cell adhesion by inside-out signaling and surface protein cleavage. Plos Biology 9. 10.1371/journal.pbio.1000587 PMC3032545 21304920

[16] Martinez-Gil M, Ramos-Gonzalez MI, Espinosa-Urgel M (2014) Roles of cyclic Di-GMP and the Gac system in transcriptional control of the genes coding for the *Pseudomonas putida* adhesins LapA and LapF. J Bacteriol 196: 1484–1495. doi: 10.1128/JB.01287-13 24488315 10.1128/JB.01287-13 PMC3993364

[17] Xiao Y, Nie H, Liu H, Luo X, Chen W, Huang Q (2016) C‐di‐GMP regulates the expression of lapA and bcs operons via FleQ in *Pseudomonas putida* KT2440. Environ Microbiol Reports 8: 659–666. 10.1111/1758-2229.12419 27120564

[18] Jimenez-Fernandez A, Lopez-Sanchez A, Jimenez-Diaz L, Navarrete B, Calero P, Platero AI, et al (2016) Complex interplay between FleQ, cyclic diguanylate and multiple sigma factors coordinately regulates flagellar motility and biofilm development in *Pseudomonas putida*. PLoS One 11. 10.1371/journal.pone.0163142 PMC5026340 27636892

[19] Yuste L, Hervas AB, Canosa I, Tobes R, Jimenez JI, Nogales J, et al (2006) Growth phase-dependent expression of the *Pseudomonas putida* KT2440 transcriptional machinery analysed with a genome-wide DNA microarray. Environ Microbiol 8: 165–177. doi: 10.1111/j.1462-2920.2005.00890.x 16343331 10.1111/j.1462-2920.2005.00890.x

[20] Jakovleva J, Teppo A, Velts A, Saumaa S, Moor H, Kivisaar M, et al (2012) Fis regulates the competitiveness of *Pseudomonas putida* on barley roots by inducing biofilm formation. Microbiology 158: 708–720. doi: 10.1099/mic.0.053355-0 22222498 10.1099/mic.0.053355-0

[21] Saldana Z, Xicohtencatl-Cortes J, Avelino F, Phillips AD, Kaper JB, Puente JL, et al (2009) Synergistic role of curli and cellulose in cell adherence and biofilm formation of attaching and effacing *Escherichia coli* and identification of Fis as a negative regulator of curli. Environ Microbiol 11: 992–1006. doi: 10.1111/j.1462-2920.2008.01824.x 19187284 10.1111/j.1462-2920.2008.01824.x PMC2672964

[22] Lahesaare A, Moor H, Kivisaar M, Teras R (2014) *Pseudomonas putida* Fis binds to the *lapF* promoter *in vitro* and represses the expression of LapF. PLoS One 9: e115901 doi: 10.1371/journal.pone.0115901 25545773 10.1371/journal.pone.0115901 PMC4278767

[23] Prigent-Combaret C, Zghidi-Abouzid O, Effantin G, Lejeune P, Reverchon S, Nasser W (2012) The nucleoid-associated protein Fis directly modulates the synthesis of cellulose, an essential component of pellicle-biofilms in the phytopathogenic bacterium *Dickeya dadantii*. Mol Microbiol 86: 172–186. doi: 10.1111/j.1365-2958.2012.08182.x 22925161 10.1111/j.1365-2958.2012.08182.x

[24] Travers A, Muskhelishvili G (2005) DNA supercoiling—a global transcriptional regulator for enterobacterial growth? Nat Rev Microbiol 3: 157–169. doi: 10.1038/nrmicro1088 15685225 10.1038/nrmicro1088

[25] Cameron AD, Stoebel DM, Dorman CJ (2011) DNA supercoiling is differentially regulated by environmental factors and FIS in *Escherichia coli* and *Salmonella enterica*. Mol Microbiol 80: 85–101. doi: 10.1111/j.1365-2958.2011.07560.x 21276095 10.1111/j.1365-2958.2011.07560.x

[26] Amzallag GN (2004) Adaptive changes in bacteria: a consequence of nonlinear transitions in chromosome topology? J Theor Biol 229: 361–369. doi: 10.1016/j.jtbi.2004.04.001 15234203 10.1016/j.jtbi.2004.04.001

[27] Gonzalez-Gil G, Bringmann P, Kahmann R (1996) FIS is a regulator of metabolism in *Escherichia coli*. Mol Microbiol 22: 21–29. 8899705 10.1111/j.1365-2958.1996.tb02652.x

[28] Bradley MD, Beach MB, de Koning AP, Pratt TS, Osuna R (2007) Effects of Fis on *Escherichia coli* gene expression during different growth stages. Microbiology 153: 2922–2940. doi: 10.1099/mic.0.2007/008565-0 17768236 10.1099/mic.0.2007/008565-0

[29] Gutierrez-Rios RM, Freyre-Gonzalez JA, Resendis O, Collado-Vides J, Saier M, Gosset G (2007) Identification of regulatory network topological units coordinating the genome-wide transcriptional response to glucose in *Escherichia coli*. BMC Microbiol 7: 53 doi: 10.1186/1471-2180-7-53 17559662 10.1186/1471-2180-7-53 PMC1905917

[30] Lenz DH, Bassler BL (2007) The small nucleoid protein Fis is involved in *Vibrio cholerae* quorum sensing. Mol Microbiol 63: 859–871. doi: 10.1111/j.1365-2958.2006.05545.x 17181781 10.1111/j.1365-2958.2006.05545.x

[31] Teras R, Jakovleva J, Kivisaar M (2009) Fis negatively affects binding of Tn*4652* transposase by out-competing IHF from the left end of Tn*4652*. Microbiology 155: 1203–1214. doi: 10.1099/mic.0.022830-0 19332822 10.1099/mic.0.022830-0

[32] Martinez-Garcia E, de Lorenzo V (2011) Engineering multiple genomic deletions in Gram-negative bacteria: analysis of the multi-resistant antibiotic profile of *Pseudomonas putida* KT2440. Environ Microbiol 13: 2702–2716. doi: 10.1111/j.1462-2920.2011.02538.x 21883790 10.1111/j.1462-2920.2011.02538.x

[33] Kivistik PA, Putrins M, Puvi K, Ilves H, Kivisaar M, Hõrak R (2006) The ColRS two-component system regulates membrane functions and protects *Pseudomonas putida* against phenol. J Bacteriol 188: 8109–8117. doi: 10.1128/JB.01262-06 17012397 10.1128/JB.01262-06 PMC1698186

[34] Miller JH (1992) A short course in bacterial genetics: a laboratory manual and handbook for *Escherichia coli* and related bacteria Cold Spring Harbor, NY: Cold Spring Harbor Laboratory Press.

[35] Sharma RC, Schimke RT (1996) Preparation of electrocompetent *E*. *coli* using salt-free growth medium. Biotechniques 20: 42–44. 8770403 10.2144/96201bm08

[36] Antoine R, Locht C (1992) Isolation and molecular characterization of a novel broad-host-range plasmid from *Bordetella bronchiseptica* with sequence similarities to plasmids from gram-positive organisms. Mol Microbiol 6: 1785–1799. 1321324 10.1111/j.1365-2958.1992.tb01351.x

[37] Kues U, Stahl U (1989) Replication of plasmids in gram-negative bacteria. Microbiol Rev 53: 491–516. 2687680 10.1128/mr.53.4.491-516.1989 PMC372750

[38] Horton RM, Hunt HD, Ho SN, Pullen JK, Pease LR (1989) Engineering hybrid genes without the use of restriction enzymes: gene splicing by overlap extension. Gene 77: 61–68. 2744488 10.1016/0378-1119(89)90359-4

[39] Choi KH, Kumar A, Schweizer HP (2006) A 10-min method for preparation of highly electrocompetent *Pseudomonas aeruginosa* cells: application for DNA fragment transfer between chromosomes and plasmid transformation. J Microbiol Methods 64: 391–397. doi: 10.1016/j.mimet.2005.06.001 15987659 10.1016/j.mimet.2005.06.001

[40] Wong SM, Mekalanos JJ (2000) Genetic footprinting with mariner-based transposition in *Pseudomonas aeruginosa*. Proc Natl Acad Sci U S A 97: 10191–10196. 10963681 10.1073/pnas.97.18.10191 PMC27802

[41] Sambrook J, Russel DW (2001) Molecular Cloning: A Laboratory Manual. New York: Cold Spring Harbor Laboratory Press.

[42] Gama-Castro S, Salgado H, Peralta-Gil M, Santos-Zavaleta A, Muniz-Rascado L, Solano-Lira H, et al (2011) RegulonDB version 7.0: transcriptional regulation of *Escherichia coli* K-12 integrated within genetic sensory response units (Gensor Units). Nucleic Acids Res 39: D98–105. doi: 10.1093/nar/gkq1110 21051347 10.1093/nar/gkq1110 PMC3013702

[43] Medina-Rivera A, Defrance M, Sand O, Herrmann C, Castro-Mondragon JA, Delerce J, et al (2015) RSAT 2015: Regulatory Sequence Analysis Tools. Nucleic Acids Res 43: W50–W56. doi: 10.1093/nar/gkv362 25904632 10.1093/nar/gkv362 PMC4489296

[44] Teras R, Hõrak R, Kivisaar M (2000) Transcription from fusion promoters generated during transposition of transposon Tn*4652* is positively affected by integration host factor in *Pseudomonas putida*. J Bacteriol 182: 589–598. 10633090 10.1128/jb.182.3.589-598.2000 PMC94319

[45] Hawley DK, McClure WR (1983) Compilation and analysis of *Escherichia coli* promoter DNA sequences. Nucleic Acids Res 11: 2237–2255. 6344016 10.1093/nar/11.8.2237 PMC325881

[46] Finkel SE, Johnson RC (1992) The Fis protein: it's not just for DNA inversion anymore. Mol Microbiol 6: 3257–3265. 1484481 10.1111/j.1365-2958.1992.tb02193.x

[47] Shao Y, Feldman-Cohen LS, Osuna R (2008) Functional Characterization of the *Escherichia coli* Fis-DNA Binding Sequence. J Mol Biol 376: 771–785. doi: 10.1016/j.jmb.2007.11.101 18178221 10.1016/j.jmb.2007.11.101 PMC2292415

[48] D'Arrigo I, Bojanovic K, Yang XC, Rau MH, Long KS (2016) Genome-wide mapping of transcription start sites yields novel insights into the primary transcriptome of *Pseudomonas putida*. Environ Microbiol 18: 3466–3481. doi: 10.1111/1462-2920.13326 27111755 10.1111/1462-2920.13326

[49] Kojic M, Venturi V (2001) Regulation of rpoS gene expression in *Pseudomonas*: involvement of a TetR family regulator. J Bacteriol 183: 3712–3720. doi: 10.1128/JB.183.12.3712-3720.2001 11371535 10.1128/JB.183.12.3712-3720.2001 PMC95248

[50] Jovcic B, Bertani I, Venturi V, Topisirovic L, Kojic M (2008) 5' Untranslated region of the *Pseudomonas putida* WCS358 stationary phase sigma factor rpoS mRNA is involved in RpoS translational regulation. J Microbiol 46: 56–61. doi: 10.1007/s12275-007-0127-2 18337694 10.1007/s12275-007-0127-2

[51] Ramos-Gonzalez MI, Molin S (1998) Cloning, sequencing, and phenotypic characterization of the rpoS gene from *Pseudomonas putida* KT2440. J Bacteriol 180: 3421–3431. 9642197 10.1128/jb.180.13.3421-3431.1998 PMC107299

[52] Ilves H, Horak R, Kivisaar M (2001) Involvement of sigma(S) in starvation-induced transposition of *Pseudomonas putida* transposon Tn*4652*. J Bacteriol 183: 5445–5448. doi: 10.1128/JB.183.18.5445-5448.2001 11514532 10.1128/JB.183.18.5445-5448.2001 PMC95431

[53] Liberati NT, Urbach JM, Miyata S, Lee DG, Drenkard E, Wu G, et al (2006) An ordered, nonredundant library of *Pseudomonas aeruginosa* strain PA14 transposon insertion mutants. Proc Natl Acad Sci U S A 103: 2833–2838. doi: 10.1073/pnas.0511100103 16477005 10.1073/pnas.0511100103 PMC1413827

[54] Mendoza-Vargas A, Olvera L, Olvera M, Grande R, Vega-Alvarado L, Taboada B, et al (2009) Genome-wide identification of transcription start sites, promoters and transcription factor binding sites in *E*. *coli*. PLoS One 4. 10.1371/journal.pone.0007526 PMC2760140 19838305

[55] Conway T, Creecy JP, Maddox SM, Grissom JE, Conkle TL, Shadid TM, et al (2014) Unprecedented high-resolution view of bacterial operon architecture revealed by RNA sequencing. Mbio 5. 10.1128/mBio.01442-14 PMC4161252 25006232

[56] Ramos-Gonzalez MI, Campos MJ, Ramos JL (2005) Analysis of *Pseudomonas putida* KT2440 gene expression in the maize rhizosphere: *in vivo* [corrected] expression technology capture and identification of root-activated promoters. J Bacteriol 187: 4033–4041. doi: 10.1128/JB.187.12.4033-4041.2005 15937166 10.1128/JB.187.12.4033-4041.2005 PMC1151710

[57] Mann EE, Wozniak DJ (2012) *Pseudomonas* biofilm matrix composition and niche biology. FEMS Microbiol Rev 36: 893–916. doi: 10.1111/j.1574-6976.2011.00322.x 22212072 10.1111/j.1574-6976.2011.00322.x PMC4409827

[58] Winsor GL, Griffiths EJ, Lo R, Dhillon BK, Shay JA, Brinkman FS (2016) Enhanced annotations and features for comparing thousands of *Pseudomona*s genomes in the *Pseudomonas* genome database. Nucleic Acids Res 44: D646–653. doi: 10.1093/nar/gkv1227 26578582 10.1093/nar/gkv1227 PMC4702867

[59] Tanaka K, Takayanagi Y, Fujita N, Ishihama A, Takahashi H (1993) Heterogeneity of the principal sigma factor in *Escherichia coli*: the *rpoS* gene product, sigma 38, is a second principal sigma factor of RNA polymerase in stationary-phase *Escherichia coli*. Proc Natl Acad Sci U S A 90: 8303 8367498 10.1073/pnas.90.17.8303a PMC55626

[60] Gaal T, Ross W, Estrem ST, Nguyen LH, Burgess RR, Gourse RL (2001) Promoter recognition and discrimination by E sigma(s) RNA polymerase. Mol Microbiol 42: 939–954. 11737638 10.1046/j.1365-2958.2001.02703.x

[61] Jyot J, Dasgupta N, Ramphal R (2002) FleQ, the major flagellar gene regulator in *Pseudomonas aeruginosa*, binds to enhancer sites located either upstream or atypically downstream of the RpoN binding site. Journal of Bacteriology 184: 5251–5260. doi: 10.1128/JB.184.19.5251-5260.2002 12218010 10.1128/JB.184.19.5251-5260.2002 PMC135358

