# Correction: The promoter region of *lapA* and its transcriptional regulation by Fis in *Pseudomonas putida*

**DOI:** 10.1371/journal.pone.0192336

**Published:** 2018-01-30

**Authors:** Hanna Ainelo, Andrio Lahesaare, Annika Teppo, Maia Kivisaar, Riho Teras

Table 1 appears incorrectly. Separately analyzed groups of data should be divided by bold horizontal lines and background color. Please see the corrected Table 1 here.

**Table 1:** B-galactosidase activity (Miller units) expressed from different length *lapA* promoter constructs.

Construct	4 h		18 h	
	PSm	PSm∆rpoS	PSm	PSm∆rpoS
pBLKT*	0.36 (0.05) a	0.58 (0.30) a	0.98 (0.05) b	1.09 (0.13) b
pB_P_lapA_1	*-*	*-*	0.8 (0.2) a	*-*
pB_P_lapA_1-2	*-*	*-*	9.9 (2.5) a	*-*
pB_P_lapA_1-3	*-*	*-*	570.6 (46.4) b	*-*
pB_P_lapA_1-4	*-*	*-*	501.5 (95.7) bc	*-*
pB_P_lapA_1-5	*-*	*-*	567.7 (21.4) bc	*-*
pB_P_lapA_1-6	*-*	*-*	861.7 (53.0) d	*-*
pB_P_lapA_1-7	*-*	*-*	457.5 (95.4) c	*-*
pB_P_lapA_1-8	*-*	*-*	516.7 (37.7) bc	*-*
pB_P_lapA_2	9.7 (1.6) a	7.7 (1.0) a	18.4 (3.3) b	16.9 (2.4) b
pB_P_lapA_2mut	7.4 (1.2) a	*-*	15.1 (3.0) b	*-*
pB_P_lapA_3	398.4 (31.2) b	480.2 (44.0) b	1217.8 (106.5) c	1443.3 (90.0) d
pB_P_lapA_3mut	28.7 (2.6) a	*-*	71.0 (4.4) a	*-*
pB_P_lapA_4	70.4 (9.0) b	67.7 (3.2) b	374.5 (74.2) c	385.5 (113.8) c
pB_P_lapA_4mut	2.4 (0.6) a	*-*	10.8 (2.1) ab	*-*
pB_P_lapA_5	20.9 (2.3) b	22.6 (4.1) b	91.2 (5.5) d	83.9 (9.0) d
pB_P_lapA_5mut	10.2 (0.8) a	*-*	38.8 (2.9) c	*-*
pB_P_lapA_6	8.1 (1.1) b	8.7 (1.3) b	34.4 (1.5) d	19.7 (1.5) c
pB_P_lapA_6mut	0.4 (0.1) a	*-*	2.4 (0.3) a	*-*
pB_P_lapA_7	8.8 (1.5) b	7.6 (0.3) b	37.6 (4.0) d	24.0 (1.5) c
pB_P_lapA_7mut	1.1 (0.1) a	*-*	2.8 (0.3) ab	*-*
pB_P_lapA_8	32.6 (2.2) b	35.9 (2.4) b	163.2 (7.5) d	123.0 (11.5) c
pB_P_lapA_8mut	12.0 (1.4) a	*-*	33.1 (7.4) b	*-*

*The promoterless pBLKT does not contain *lapA* upstream DNA.

Data from at least 5 independent measurements is shown. 95% confidence intervals are shown in parentheses. Not determined activities are marked with the “-”symbol. Groups of data analysed separately are divided by bold horizontal lines and background colour. Letters a-e depict different homogeneity groups according to ANOVA *post hoc* Bonferroni test. Identical letters denote non-significant differences (*P*>0.05) between averages of β-galactosidase activity.
