# Supplementary material for: The promoter region of lapA and its transcriptional regulation by Fis in Pseudomonas putida
Source: PLoS One. 2017 Sep 25;12(9):e0185482. doi: 10.1371/journal.pone.0185482 (PMC5612765; doi:10.1371/journal.pone.0185482)
Supplement: S1 Table — (DOCX) [file pone.0185482.s001.docx]

**S1 Table.** **Bacterial strains and plasmids used in this study**

| **Strain and plasmid** | **Genotype or description** | **Source/ reference** |
| --- | --- | --- |
| ***E. coli*** |  |  |
| DH5*αλpir* | *λpir* lysogen of DH5*α* | [[32](#_ENREF_32)] |
| ***P. putida*** |  |  |
| PSm | PaW85 (isogenic to KT2440); chromosomal mini-Tn*7*-ΩSm1 (Sm^r^) | [[20](#_ENREF_20)] |
| PSm Δ*rpoS* | PSm; Δ*rpoS* (Sm^r^) | This study |
| F15 | PaW85 (isogenic to KT2440); chromosomal mini-Tn*7*-ΩGm-term-*lacI*^q^-P*_tac_*-*fis*-T1T2 (Gm^r^) | [[20](#_ENREF_20)] |
| **Promoter-probe vectors** |  |  |
| pBLKT | A promoter probe vector containing *lacZ* reporter gene (Km^r^), pBBR1-based vector | [[22](#_ENREF_22)] |
| p9TT_B_lacZ | A promoter probe vector containing *lacZ* reporter gene (Amp^r^); RK2-based vector | [[33](#_ENREF_33)] |
| **pBLKT vectors with *lapA* promoter region** | **Length of cloned fragment; primers used to amplify the fragment; potential promoters ; potential Fis binding sites** |  |
| pB_P_lapA_1 | 45 bp; LapA-I-rev and LapA-II; potential promoter P*_lapA1_*; no potential Fis binding sites | This study |
| pB_P_lapA_1-2 | 131 bp; LapA-I-rev and LapA-III; potential promoters P*_lapA1_* and P*_lapA2_*; potential Fis binding sites Fis-A1 and Fis-A2 | This study |
| pB_P_lapA_1-3 | 173 bp; LapA-I-rev and LapA-IV; potential promoters P*_lapA1_* to P*_lapA3_*; potential Fis binding sites Fis-A1 and Fis-A2 | This study |
| pB_P_lapA_1-3_P_lapA_3mut | 173 bp; LapA-I-rev and LapA-IV; mutated -10 box of potential promoter P*_lapA3_*; potential promoters P*_lapA1_* and P*_lapA2_*; potential Fis binding sites Fis-A1 and Fis-A2 | This study |
| pB_P_lapA_1-4 | 237 bp; LapA-I-rev and LapA-V; potential promoters P*_lapA1_* to P*_lapA4_*; potential Fis binding sites Fis-A1 to Fis-A3 | This study |
| pB_P_lapA_1-5 | 362 bp; LapA-I-rev and LapA-VI; potential promoters P*_lapA1_* to P*_lapA5_*; potential Fis binding sites Fis-A1 to Fis-A3 | This study |
| pB_P_lapA_1-6 | 475 bp; LapA-I-rev and LapA-fw; potential promoters P*_lapA1_* to P*_lapA6_*; potential Fis binding sites Fis-A1 to Fis-A4 | This study |
| pB_P_lapA_1-7 | 571 bp; LapA-I-rev and LapA-VIII; potential promoters P*_lapA1_* to *_PlapA7_*; potential Fis binding sites Fis-A1 to Fis-A5 | This study |
| pB_P_lapA_1-8 | 951 bp; LapA-I-rev and PP0167-down; potential promoters P*_lapA1_* to P*_lapA8_*; potential Fis binding sites Fis-A1 to Fis-A8 | This study |
| pB_P_lapA_2 | 88 bp; LapA-II-rev and LapA-III; potential promoter P*_lapA2_*; potential Fis binding sites FisA1 and FisA2 | This study |
| pB_P_lapA_2mut | 88 bp; A2prom-mut and LapA-III; mutated potential promoter P*_lapA2_*; potential Fis binding sites FisA1 and FisA2 | This study |
| pB_P_lapA_3 | 43 bp, LapA-III-rev and LapA-IV; potential promoter P*_lapA3_*; no potential Fis binding sites | This study |
| pB_P_lapA_3mut | 43 bp, A3prom-mut and LapA-IV; mutated potential promoter P*_lapA3_*; no potential Fis binding sites | This study |
| pB_P_lapA_4 | 64 bp; LapA-IV-rev and LapA-V; potential promoter P*_lapA4_*; potential Fis binding site Fis-A3 | This study |
| pB_P_lapA_4mut | 64 bp; A4prom-mut and LapA-V; mutated potential promoter P*_lapA4_*; potential Fis binding site Fis-A3 | This study |
| pB_P_lapA_5 | 126 bp; LapA-V-rev and LapA-VI; potential promoter P*_lapA5_*; no potential Fis binding sites | This study |
| pB_P_lapA_5mut | 126 bp; A5prom-mut and LapA-VI; mutated potential promoter P*_lapA5_*; no potential Fis binding sites | This study |
| pB_P_lapA_6 | 116 bp; LapA-VI-rev and LapA-fw; potential promoter P*_lapA6_*; partial Fis binding site Fis-A4 | This study |
| pB_P_lapA_6mut | 116 bp; A6prom-mut and LapA-fw; mutated potential promoter P*_lapA6_*; potential Fis binding site Fis-A4 | This study |
| pB_P_lapA_6B | 144 bp; LapA-VI-rev2 and LapA-fw; potential promoter P*_lapA6_*; potential Fis binding site Fis-A4 | This study |
| pB_P_lapA_6B_F4mut | 144 bp; LapA-VI-rev2 and LapA-fw; potential promoter P*_lapA6_*; nine substitutions in the Fis binding site Fis-A4 | This study |
| pB_P_lapA_7 | 66 bp; LapA-VII-rev and LapA-VIII; potential promoter P*_lapA7_*; potential Fis binding site Fis-A5 | This study |
| pB_P_lapA_7mut | 66 bp; A7prom-mut and LapA-VIII; mutated potential promoter P*_lapA7_*; potential Fis binding site Fis-A5 | This study |
| pB_P_lapA_8 | 105 bp; LapA-VIIIrev and PP0168-I-fw; potential promoter P*_lapA8_*; potential Fis binding site Fis-A6 | This study |
| pB_P_lapA_8mut | 105 bp; A8prom-mut and PP0168-I-fw; mutated potential promoter P*_lapA8_*; potential Fis binding site Fis-A6 | This study |
| **p9TT_B_lacZ vectors with *lapA* promoter region** | **Length of cloned fragment; primers used to amplify the fragment; potential promoters ; potential Fis binding sites** |  |
| p9_P_lapA_1-8 | 951 bp; LapA-I-rev and PP0167-down; potential promoters P*_lapA1_* to P*_lapA8_*; potential Fis binding sites Fis-A1 to Fis-A8 | This study |
| p9_ P_lapA_1-8_F1mut | 951 bp; LapA-1mut-uus and LapA-I-rev and in second PCR PP0167-down, potential promoters P*_lapA1_* to P*_lapA8_*; potential Fis binding sites Fis-A1 to Fis-A8 (six substitutions in Fis-A1) | This study |
| p9_ P_lapA_1-8_F2mut | 951 bp; LapA-2mut and LapA-I-rev and in second PCR PP0167-down; potential promoters P*_lapA1_* to P*_lapA8_*; potential Fis binding sites Fis-A1 to Fis-A8 (four substitutions in Fis-A2) | This study |
| p9_ P_lapA_1-8_F1,2mut | 951 bp; LapA-I-rev and PP0167-down; potential promoters P*_lapA1_* to P*_lapA8_*; potential Fis binding sites Fis-A1 to Fis-A8 (six substitutions in Fis-A1 and four substitutions in Fis-A2) | This study |
| p9_ P_lapA_1-8_F4mut | 951 bp; LapA-4mut-uus and PP0167-down and in second PCR PP0167-down; potential promoters P*_lapA1_* to P*_lapA8_*; potential Fis binding sites Fis-A1 to Fis-A8 (nine substitutions in Fis-A4) | This study |
| p9_ P_lapA_1-8_F5mut | 951 bp; FisA5-mut and PP0167-down and in second PCR PP0167-down; potential promoters P*_lapA1_* to P*_lapA8_*; potential Fis binding sites Fis-A1 to Fis-A8 (five substitutions in Fis-A5) | This study |
| p9_ P_lapA_1-8_F6mut | 951 bp; FisA6-mut and PP0167-down and in second PCR PP0167-down; potential promoters P*_lapA1_* to P*_lapA8_*; potential Fis binding sites Fis-A1 to Fis-A8 (six substitutions in Fis-A6) | This study |
| p9_ P_lapA_1-8_F7mut | 951 bp; FisA7-mut and PP0167-down and in second PCR PP0167-down; potential promoters P*_lapA1_* to P*_lapA8_*; potential Fis binding sites Fis-A1 to Fis-A8 (six substitutions in Fis-A7) | This study |
| p9_ P_lapA_1-8_F4,5,6,7mut | 951 bp; LapA-I-rev and PP0167-down; potential promoters P*_lapA1_* to P*_lapA8_*; potential Fis binding sites Fis-A1 to Fis-A8 (nine substitutions in Fis-A4, five in Fis-A5, six in Fis-A6 and six in Fis-A7) | This study |
| p9_P_lapA_6B | 144 bp; LapA-VI-rev2 and LapA-fw; potential promoter P*_lapA6_*; potential Fis binding site Fis-A4 | This study |
| p9_P_lapA_6B_F4mut | 144 bp; LapA-VI-rev2 and LapA-fw; potential promoter P*_lapA6_*; nine substitutions in the Fis binding site Fis-A4 | This study |
| p9_P_lapA_7 | 66 bp; LapA-VII-rev and LapA-VIII; potential promoter P*_lapA7_*; potential Fis binding site Fis-A5 | This study |
| p9_P_lapA_7_F5mut | 66 bp; LapA-VII-rev and LapA-VIII-mut; potential promoter P*_lapA7_*; five substitutions in the Fis binding site Fis-A5 | This study |
| p9_P_lapA_8B | 381 bp; LapA-VIII-rev and PP0168-down; potential promoter P*_lapA8_*; potential Fis binding sites Fis-A6 and Fis-A7 | This study |
| p9_P_lapA_8B_F6mut | 381 bp; LapA-VIII-rev-mut and PP0168-down; potential promoter P*_lapA8_*; potential Fis binding site Fis-A7 (six substitutions in Fis-A6) | This study |
| p9_P_lapA_8B_F7mut | 381 bp; LapA-VIII-rev and PP0168-down; potential promoter P*_lapA8_*; potential Fis binding site Fis-A6 (six substitutions in Fis-A7) | This study |
| p9_P_lapA_8B_F6,7mut | 381 bp; LapA-VIII-rev-mut and PP0168-down; potential promoter P*_lapA8_*; six substitutions in the Fis binding site Fis-A6 and six substitutions in Fis-A7 | This study |
| **Other plasmids** |  |  |
| pEMG | Suicide vector for knockout mutagenesis (Km^r^) | [[32](#_ENREF_32)] |
| pEMG-Δ*rpoS* | A PCR-fragment containing 457 bp of upstream and 468 bp of downstream DNA of the *rpoS* gene cloned into pEMG opened with EcoRI and BamHI (Km^r^) | This study |
| pSW (Sce-I) | I-SceI-expressing plasmid (Amp^r^) | [[40](#_ENREF_40)] |
| pLA1-12 | Carrying LF2 site in left end DNA of Tn*4652*; (Amp^r^) | [[44](#_ENREF_44)] |
| pRA1-12 | Carrying RF1 site in right end DNA of Tn*4652*; (Amp^r^) | [[44](#_ENREF_44)] |
