# Supplementary material for: The promoter region of lapA and its transcriptional regulation by Fis in Pseudomonas putida
Source: PLoS One. 2017 Sep 25;12(9):e0185482. doi: 10.1371/journal.pone.0185482 (PMC5612765; doi:10.1371/journal.pone.0185482)
Supplement: S2 Table — a restrictases are shown in brackets. b sequences recognized by endonucleases are underlined, the nucleotides mutated in Fis binding sites or -10 boxes are shown in bold and sequences complementary to another oligonucleotide are indicated in bold and italics. (DOCX) [file pone.0185482.s002.docx]

**S2 Table. Oligonucleotides used in this study**

| **Oligonucleotide designation*^a^*** | **Oligonucleotide sequence 5’-3’*^b^*** | **Use** |
| --- | --- | --- |
| A2prom-mut (BamHI) | TTGGATCCTATGTCG**CG**G**CG**CTCTTGACGTTTTTG | Construction of pB_P_lapA_2mut |
| A3prom-mut (BamHI) | TTGGATCCTACATGCC**CG**C**CC**GAACAATCGGC | Construction of pB_P_lapA_3mut |
| A4prom-mut (BamHI) | TTGGATCCTATTTGT**G**CC**GCC**GCCAAGCGTTTC | Construction of pB_P_lapA_4mut |
| A5prom-mut (BamHI) | TTGGATCCAGCCATCAG**G**CC**GC**GCACTTTTTC | Construction of pB_P_lapA_5mut |
| A6prom-mut (BamHI) | TTGGATCCTGATTGCC**G**A**G**T**CC**GCACAAACCG | Construction of pB_P_lapA_6mut |
| A7prom-mut (BamHI) | TTGGATCCGTGAATA**GC**GG**CC**TCCCGATCATTG | Construction of pB_P_lapA_7mut |
| A8prom-mut (BamHI) | TTGGATCCGCACTC**CGC**AGATGAATTTTGAC | Construction of pB_P_lapA_8mut |
| Adapt-lyh | GACTCGAGTCGACATCG | Adaptor primer, identical to 5ʹ end of Adapt-pikk oligonucleotide |
| Adapt-pikkC | GACTCGAGTCGACATCGA(C)_17_ | Synthesis of the second cDNA strand in RACE, binds poly-G |
| Adapt-pikkT | GACTCGAGTCGACATCGA(T)_17_ | Synthesis of the second cDNA strand in RACE, binds poly-A |
| FisA5-mut | AAATCGCTTGCGGA**GGC**TGA**C**TG**GG**AGAAGGCAAGAATC | Construction of p9_ P_lapA_1-8_F5mut and p9_ P_lapA_1-8_F4,5,6,7mut |
| FisA6-mut | ATCCGCACTCTATA**C**A**C**G**T**A**GGCC**GACTAACAAGCCACA | Construction of p9_ P_lapA_1-8_F6mut and p9_ P_lapA_1-8_F4,5,6,7mut |
| FisA7-mut | TGATGGCTGGATCA**GGCC**TG**TC**A**G**AATCATTGCCCCGCT | Construction of p9_ P_lapA_1-8_F7mut and p9_ P_lapA_1-8_F4,5,6,7mut |
| LacZ-AB | GTGTTACCCAGAGTCAGC | Verification and sequencing of pBLKT constructs |
| LapA2up | AGGATCCGAAACGCTTGGCTAAGGTA | Amplification of the fragment containing Fis-A1 and Fis-A2 binding sites for DNase I footprint |
| LapAdown | GGTACTTCCCTGCCAGAG | Amplification of the fragment containing Fis-A1 and Fis-A2 binding sites for DNase I footprint |
| LapAdown2 | CACCTCAGCAGCCTGAAATCA | Amplification of the fragment containing Fis-A4 binding site for DNase I footprint |
| LapA-FisA1mut-uus | CGGAATATTGACGTCA**CCC**ACGT**GCC**GAGATCATCGAC | Construction of p9_ P_lapA_1-8_F1mut and p9_ P_lapA_1-8_F1,2mut |
| LapA-FisA2mut | ATGGCATGTATAAG**C**T**G**AATAGTTTGG**AG**GTCAGGCAAT | Construction of p9_ P_lapA_1-8_F2mut and p9_ P_lapA_1-8_F1,2mut |
| LapA-FisA4mut-uus | CGCCCGGACAC**GA**ATT**C**CC**G**A**G**T**CC**G**TC**CAAACCTTGCC | Construction of p9_ P_lapA_1-8_F4mut and p9_ P_lapA_1-8_F4,5,6,7mut |
| LapA-fw (BamHI) | TGGATCCACATAGCTGCTTGTGACTCG | Construction of pB_P_lapA_1-6, pB_P_lapA_6 and pB_P_lapA_6mut Amplification of the fragment containing Fis-A4 binding site for DNase I footprint |
| LapA-II (BamHI) | GGGATCCGTTCCGCCTGAAGTGGC | Construction of pB_P_lapA_1 |
| LapA-III (BamHI) | GGGATCCGTATAAGGTCAATAGTTTGGC | Construction of pB_P_lapA_1-2, pB_P_lapA_2 and pB_P_lapA_2mut |
| LapA-IIImut-pikem | CTTATACATGCC**CG**CC**CG**A | Construction of pB_P_lapA_1-3_ P_lapA_3mut |
| LapA-III-rev (BamHI) | AAGGATCCTACATGCCATCCTAAAC | Construction of pB_P_lapA_3 |
| LapA-II-rev (BamHI) | AAGGATCCTATGTCGATGATCTCTTGA | Construction of pB_P_lapA_2 |
| LapA-I-rev (BamHI) | AAGGATCCTACTTCCCTGCCAGAGCG | Construction of pB_P_lapA_1, pB_P_lapA_1-2, pB_P_lapA_1-3, pB_P_lapA_1-4, pB_P_lapA_1-5, pB_P_lapA_1-6, pB_P_lapA_1-7, pB_P_lapA_1-8 and p9_P_lapA_1-8 variants of p9_ P_lapA_1-8 with mutated Fis binding sites |
| LapA-IV (BamHI) | AGGATCCATCAATGTGACATTACATTGC | Construction of pB_P_lapA_3, pB_P_lapA_3mut, pB_P_lapA_1-3 and pB_P_lapA_1-3_ P_lapA_3mut |
| LapA-IV-rev (BamHI) | AAGGATCCTATTTGTACCTTAGCCAAGC | Construction of pB_P_lapA_4 and amplification two-stranded cDNA in RACE |
| LapA-RACE1 | AGCTCAGTAGTCGGGTCAA | Synthesis of cDNA first strand in RACE |
| LapA-RACE2 | CATCAGCCAGCTCCAAAGT | Amplification of two-stranded cDNA in RACE |
| LapA-rev (BamHI) | TGGATCCGACAATGCTTTTGACGATGG | Amplification of two-stranded cDNA in RACE |
| LapA-V (BamHI) | AGGATCCTTAACACCTTTTTGATGATG | Construction of pB_P_lapA_1-4, pB_P_lapA_4, pB_P_lapA_4mut |
| LapA-VI (BamHI) | AGGATCCAGTGTCCGGGCGATTGAC | Construction of pB_P_lapA_1-5, pB_P_lapA_5 and pB_P_lapA_5mut |
| LapA-VIII (BamHI) | TTGGATCCGATTCTTGCCTTCTTGCATT | Construction of pB_P_lapA_1-7, pB_P_lapA_7, pB_P_lapA_7mut and p9_ P_lapA_7 |
| LapA-VIII-mut (BamHI) | TTGGATCCGATTCTTGCCTTCT**CC**CA**G**T | Construction of p9_P_lapA_7_F5mut |
| LapA-VIII-rev (BamHI) | AAGGATCCGCACTCTATAGATGAATT | Construction of pB_P_lapA_8, p9_P_lapA_8B and p9_P_lapA_8B_F7mut |
| LapA-VIII-rev-mut (BamHI) | AAGGATCCGCACTCTATA**C**A**C**G**T**A**GG** | Construction of p9_P_lapA_8B_F6mut and p9_P_lapA_8B_F6,7mut |
| LapA-VII-rev (BamHI) | AAGGATCCGTGAATAATGGTATCCCG | Construction of pB_P_lapA_7, p9_P_lapA_7 and p9_P_lapA_7_F5mut |
| LapA-VI-rev (BamHI) | AAGGATCCTGATTGCCAAATAAGCACA | Construction of pB_P_lapA_6 and amplification of two-stranded cDNA in RACE |
| LapA-VI-rev2 | AAGGATCCAGGTCACTGTCAATCG | Construction of pB_P_lapA_6B and pB_P_lapA_6B_F4mut |
| LapA-V-rev (BamHI) | AAGGATCCAGCCATCAGTCCTAGCAC | Construction of pB_P_lapA_5 |
| LapBCdown | TGCTGATAAGGGTTATCAC | Amplification of the fragment containing Fis-A7 binding site for DNase I footprint |
| LapBCup (BamHI) | TGGATCCAGCGCCTGTTTACTGACA | Construction of pB_Ataga , amplification of the fragment containing Fis-A7 binding site for DNase I footprint |
| PP0167-down (BamHI) | TTTGGATCCGCGGCGCTCTCTTCATCC | Construction of pB_P_lapA_1-8, p9_P_lapA_1-8 and p9_ P_lapA_1-8 variants containing mutations in Fis binding site(s); p9_ P_lapA_8B and p9_ P_lapA_8B variants with mutated Fis-A6 and/or Fis-A7 binding sites |
| PP0167-I-fw (BamHI) | TTTGGATCCGAGTCACAAGCAGCTATGT | Amplification of the fragment containing Fis-A5 and Fis-A6 binding sites for DNase I footprint |
| PP0168-I-fw (EcoRI) | TTGAATTCATGCAAGGGCTTTGGGTTC | Construction of pB_P_lapA_8 andpB_P_lapA_8mut; amplification of the fragment containing Fis-A6 binding sites for DNase I footprint |
| PRH8 | GCTGAGCTCAGACGGTGGATGACCAGC | Amplification of RF1 for gel mobility shift assay |
| rpoS-2-fw | GGTCTGTCCAGCGAGTCAT | Construction of pEMG-ΔrpoS |
| rpoS-2-rev (BamHI) | AAGGATCCTTACTACGGCTTCGGCTG | Construction of pEMG-ΔrpoS |
| rpoS-I-fw (EcoRI) | AAGAATTCAACGGTGTACTGATTGGAA | Construction of pEMG-ΔrpoS |
| rpoS-I-rev | ***ATGACTCGCTGGACAGACC***TGTTATAATCCTTTGCTGAG | Construction of pEMG-ΔrpoS |
| rpoS-kesk | GGCAATTTTGACAACCAGGC | Verification of *rpoS* deletion in *P. putida* chromosome |
| rpoS-taga | GGCATAACCTGTCGGC | Verification of *rpoS* deletion in *P. putida* chromosome |
| rpoS-up (BamHI) | TGGATCCGTTGCTACCGTACCAGCC | Verification of *rpoS* deletion in *P. putida* chromosome codon of the *rpoS* gene (PP1623) |
| SIDD-2 | AGAGCTCCTGTACGTGCGCTT | Amplification of LF2 for gel mobility shift assay |
| T1T2 | TGGCCTTTTTGCGTAGATC | Verification and sequencing of pBLKT constructs |
| TnLsisse | GCAAAGACTGCTTCGCGCCC | Amplification of LF2 for gel mobility shift assay |
| Tnots | GGGGTTATGCCGAGATAAGGC | Amplification of RF1 for gel mobility shift assay |

*^a^* restrictases are shown in brackets

*^b^* sequences recognized by endonucleases are underlined, the nucleotides mutated in Fis binding sites or -10 boxes are shown in bold and sequences complementary to another oligonucleotide are indicated in bold and italics.
