# Supplementary material for: The promoter region of lapA and its transcriptional regulation by Fis in Pseudomonas putida
Source: PLoS One. 2017 Sep 25;12(9):e0185482. doi: 10.1371/journal.pone.0185482 (PMC5612765; doi:10.1371/journal.pone.0185482)
Supplement: S3 Table — Data from at least 5 independent measurements is shown. 95% confidence intervals are shown in parentheses. Letters a-c depict different homogeneity groups according to ANOVA post hoc Bonferroni test. Identical letters denote non-significant differences (P>0.05) between averages of β-galactosidase activity. (DOCX) [file pone.0185482.s003.docx]

**S3 Table. B-galactosidase activity (Miller units) in *P. putida* strains PSm and F15 harbouring promoterless p9TT_B_lacZ.**

|  | PSm | | F15 | |
| --- | --- | --- | --- | --- |
|  | without IPTG | 1 mM IPTG | without IPTG | 1 mM IPTG |
| 4 h | 0.21 (0.06) a | 0.32 (0.08) ab | 0.42 (0.07) bc | 0.40 (0.04) bc |
| 18 h | 0.24 (0.04) a | 0.25 (0.04) a | 0.45 (0.07) c | 0.22 (0.05) a |
